# A multiplex microsatellite set for non-invasive genotyping and sexing of the osprey (*Pandion haliaetus*)

**DOI:** 10.1007/s12686-015-0497-4

**Published:** 2015-10-29

**Authors:** Deborah A. Dawson, Oddmund Kleven, Natalie dos Remedios, Gavin J. Horsburgh, Rolf T. Kroglund, Teresa Santos, Colin R. A. Hewitt

**Affiliations:** Department of Animal and Plant Sciences, University of Sheffield, Western Bank, Sheffield, South Yorkshire, S10 2TN UK; Norwegian Institute for Nature Research (NINA), 7485 Trondheim, Norway; Nord-Trøndelag University College, 7729 Steinkjer, Norway; Department of Biology, University of Aveiro, 3810-193 Aveiro, Portugal; Department of Genetics, University of Leicester, Leicester, LE1 7RH UK

**Keywords:** Birds of prey, Feather, Raptor, Sex-typing, Simple Tandem Repeat (STR), Swabs, Western osprey

## Abstract

During the 1950s and 1970s the osprey (*Pandion haliaetus*) experienced a dramatic population crash and remains of conservation concern in several parts of the world. We isolated 37 microsatellite loci and assessed these in ospreys sampled in the UK and Norway (using mouth swabs/feathers). From 26 loci variable in four ospreys, we selected 13, combined these into two multiplex-PCR sets and included a sex-typing marker. Additional markers confirmed sexes. In 17 ospreys, feather-sampled in central Norway, we found 3–10 alleles per locus. The 13 loci are autosomal (heterozygotes were present in both sexes) and observed heterozygosities ranged from 0.24 to 0.94. The combined probability of identity for the 13 loci was 8.0 × 10^−12^. These microsatellite loci will be useful for genetic monitoring, parentage analysis and population genetic studies of the osprey.

**Electronic supplementary material:**

The online version of this article (doi:10.1007/s12686-015-0497-4) contains supplementary material, which is available to authorized users.

## Introduction

The osprey (*Pandion haliaetus*) is a fish-eating raptor with an almost worldwide distribution. It experienced a dramatic decline in population size in the 1950s–1970s primarily due to the use of pesticides and is studied as a sentinel species to detect pollution (Grove et al. 2009). European populations of ospreys are migratory, spending the summer in Europe and winter in Africa, whereas other populations are resident. Although the osprey has recovered to some degree and is no longer threatened globally, it is still of conservation concern in some areas (BirdLife International 2013). To facilitate genetic monitoring through non-invasive sampling of shed feathers, and to enable analyses of genetic diversity, parentage and population structure, we isolated and characterized novel microsatellite loci for the osprey.

## Methods

Microsatellite sequences were isolated from a male osprey (02/09). This individual hatched at Rutland Water Nature Reserve, near Oakham, UK in 2009 but died of an infection at 6 weeks old. Genomic DNA was extracted from liver tissue, digested with *Mbo*I, enriched for dinucleotide/tetranucleotide sequences, cloned and Sanger-sequenced bidirectionally, identifying 96 unique osprey microsatellites (following Armour et al. 1994). In addition, an Illumina paired-end library was created from the dinucleotide + tetranucleotide-enriched DNA (~1 + 1 µg) and MiSeq-sequenced. This allowed more (tetranucleotide) marker choices for multiplexing. Primer sets were designed from 37 sequences (26 Sanger and 11 MiSeqs) using Primer3 v0.4.0.

Samples were collected from wild ospreys including: (1) 17 feathers from nine nests in central Norway (two plucked from unrelated nestlings and 15 shed from adults); (2) six feathers from two nests in Scotland; and 3) mouth-swabs from 48 osprey chicks at Rutland Water Nature Reserve, England. For genotyping, DNA was extracted from feather calamus (‘Norwegian’ ospreys) using the Maxwell^®^16 Research System (Promega), and from feathers (‘Scottish’ ospreys) or mouth swabs (‘English’ chicks) using ammonium acetate. We sexed the chick and feather samples using the *Z*-*002A*, *Z*-*002D* (Dawson 2007) and *Z43B* markers (DAD et al. unpublished data). Initially, each locus was amplified in ospreys sampled in Norway (n = 4), Scotland (n = 6) and England (n = 48; Table 1). PCR was performed with fluorescently-labeled forward primers using QIAGEN’s Multiplex PCR kit and protocol [annealing temperature = 56/57 °C (Table 1); 2/10-µl reactions]. Multiplex-PCR was used to genotype/sex-type the 17 presumably unrelated ospreys, sampled in Nord-Trondelag county (64°06′N, 12°50′E), central Norway (Table 2). PCR products were separated on an ABI Genetic Analyzer and allele sizes assigned using Genemapper software.

**Table 1:** Assessment of 37 osprey (*Pandion haliaetus*) microsatellite loci in three populations

Locus^a^	Clone name and ENA sequence accession no.	Chromosome location^b^	Primer sequences (5′–3′)		Primer Tm (°C)^c^	Repeat motif	Country where sampled	n	A	Expected allele size (bp)^d^	Observed allele size range (bp)
Pha01	Osp107_A01WZ	Gga and Tgu,	F:	[HEX]GTCAACAGTGTGCCCTAGCAG	60.90	(TG)_10_	NOR	4	0	195	No amp.
Unreliable	LN829364	Multiple copies	R:	TACCCGGGAAGCTTGGAC	61.00		SCOT	6	0	Unreliable	Poor amp.
		NCBI: GgaW & Z					ENG	48	0		No amp.
**Pha02**	Osp107_A08	Gga6, 26441864	F:	Set 1 = [PET]TTATCTGCAAGGCCTGGTGG	63.37	(CA)_15_	NOR	4	5	200	196–208
	LN829365	Tgu6, 24709071	R:	ACAGGAGTGGAGGAGGTAGT	55.22						
			F:	Set 2 (UK) = [6FAM]ATTATCTGCAAGGCCTGGTG	60.10		SCOT	4	3	256	253–259
			R:	CTGCTGCTTGGAAATGCTC	59.69		ENG	48	4		253–261
Pha03	Osp107_C03	Gga17, 5182075	F:	[6FAM]TCTAGCCCATCTCCAGTGAATC	59.03	(TG)_9_	NOR	4	2	112	105–111
	LN829366	Tgu17, 5717909	R:	AATTAGAAAGTTGGTGCAGTCCC	59.17		SCOT	6	1		111
							ENG	48	1		111
Pha04	Osp107_C09	Gga3, 45456042	F:	[VIC]ATGACCAGTCTGATGCCTTG	58.67	(CA)_12_	NOR	4	4	160	158–168
	LN829367	Tgu3, 39010319	R:	ACATTTGGAGGGTTTCTTGC	59.03						
			F:	[HEX] used for UK samples			SCOT	6	5	160	143–167
							ENG	48	6		143–165
Pha05	Osp107_C10	Gga3, 21714508	F:	[HEX]CATTTAACGGTTTAGAAAGTGAAGG	59.54	(GT)_12_	NOR	4	2	259	259–261
	LN829368	Tgu3, 11588718	R:	TGTAGTGAAATGAATAACAAATGAAGC	59.87		SCOT	6	2		248–261
							ENG	48	2		259–261
Pha06	Osp107_D06	Gga1, 182509839	F:	[HEX]CAAGCTTGTAGCAGTCTTGCAG	60.38	(CA)_19_	NOR	4	0	117	No amp.
	LN829369	Tgu1, 4702446	R:	TGCCTGTACAGAAGCAGCAG	60.35		SCOT	6	3		108–113
							ENG	48	2		108–112
Pha07	Osp107_D07	Gga9, 5205116	F:	[6FAM]GATCACCTCGCTCATCTAG	54.30	(AC)_9_	NOR	4	1	125	122
	LN829370	Tgu9, 841456	R:	ACGTAACTAAAGAGAGCCTC	54.25		SCOT	6	1		121
							ENG	47	1		121
Pha08	Osp107_D08	Gga7, 27710560	F:	[HEX]TACAGGGAGGTCAGCCAATC	60.07	(AC)_12_	NOR	4	0	209	No amp.
Unreliable	LN829371	Tgu7, 6096203	R:	GGGTTTGCCTACATGGGTATC	60.45		SCOT	4	3	Unreliable	(201–211)
							ENG	48	0		No amp.
Pha09	Osp107_F09	Gga4, 60184616	F:	[6FAM]CTTGCTGCCAGTTGCTAGG	59.75	(TG)_11_	NOR	4	2	248	258–261
	LN829372	Tgu4, 19049909	R:	TTAGGGAAGGCAGTTGATGAG	59.32		SCOT	6	2		250–252
							ENG	48	2		250–252
**Pha10**	Osp107_F12	Gga—no hits	F:	Set 1 = [PET]TGGTGAGAAGCCCAGTGAAA	61.78	(GT)_22_	NOR	4	4	178	183–211
	LN829373	Tgu3, 76487951	R:	ACATTACCCTTCACCTTGTTCA	58.49						
			F:	Set 2 (UK) = [6FAM]GAAGCCCAGTGAAAGTAAGATAGG	59.70		SCOT	6	5	299	300–332
			R:	GTCAGTGAAGGTGGCACAAG	59.31		ENG	47	6		300–330
Pha11	Osp107_G04	Gga26, 3808355	F:	[HEX]ATCATTGTCTCCGTTGAAATACTC	58.59	(TG)_12_	NOR	4	4	369	362–374
	LN829374	Tgu—no hits	R:	TGGCTTAAGGACATGAGCTG	59.02		SCOT	5	3		366–372
							ENG	47	4		366–374
Pha12	Osp107_G05Z	Gga—no hits	F:	[HEX]TGCATCCTAATGAACCTTTGC	60.09	(CA)_15_	NOR	4	3	299	294–302
	LN829375	TguZ, 23578707	R:	AGGCTGGTGGTTAAACATGG	59.85		SCOT	4	3	(females=	300–304
							ENG	48	3	homozyg)	300–304
**Pha13**	Osp107_G06	Gga12, 12834746	F:	[6FAM]AGACAAATTACTTTCTGCCCTGC	59.49	(AC)_9_	NOR	4	5	193	184–194
	LN829376	Tgu12, 13613680	R:	CATAGCTGCACATGACTTCCC	59.05		SCOT	6	5		185–195
							ENG	48	7		181–195
Pha14	Osp107_G07	Gga6, 7231355	F:	[6FAM]CTGAGCCCTACAGGTCAAGG	59.86	(CA)_14_	NOR	4	3	163	155–163
	LN829377	Tgu6random, 1131071	R:	GATCAAAGTATAAGCTTCTGGCACT	59.42		SCOT	6	2		155–163
							ENG	48	4		155–163
Pha15	Osp107_H11	Gga—no hits	F:	[6FAM]AGGAGAACTGGGCTTGGTC	59.24	(GT)_11_	NOR	4	2	148	149–151
	LN829378	TguLGE11random, 434714	R:	TTTGTCACTCTGAACCCAACTC	59.23		SCOT	6	2		149–151
							ENG	48	3		147–151
**Pha16**	Osp108_C02	Gga4, 60893985	F:	[6FAM]TTTAGGACATGAAAGACCATCTAGC	60.04	(GT)_11_	NOR	4	3	300	296–302
	LN829379	Tgu4, 19753992	R:	AGGCTCGAATCAAGGAATAGG	59.70		SCOT	6	4		296–302
							ENG	48	3		298–302
Pha17	Osp108_D06	Gga3, 6186457	F:	[6FAM]GATCATTTGAGTCAGGGTTGTAGA	59.53	(GT)_12_	NOR	4	2	273	258–261
	LN829380	Tgu3, 23071942	R:	CCCAGGCAATGTGTGATAGTAG	59.52		SCOT	6	4		258–263
							ENG	48	2		257–260
Pha18	Osp108_D09	Gga14, 7369333	F:	[6FAM]TTGGTCACTTCTGTGGAACC	58.54	(CT)_13_	NOR	4	6	204	205–257
	LN829381	Tgu14, 16292216	R:	GGACGCATGGTGTAAACTTC	58.08		SCOT	6	5		205–261
							ENG	47	7		205–285
Pha19	Osp108_E06	Gga2, 137582088	F:	[6FAM]ATGGTGTCGTGGTGACTGC	60.62	(GT)_11_	NOR	4	3	94	90–94
	LN829382	Tgu2, 138654459	R:	AAGCGATTCACTCCATGCTC	60.37		SCOT	6	2		90–92
							ENG	48	2		92–94
Pha20	Osp108_F01	Gga7, 32493856	F:	[HEX]CTTTGTGAGCCTGCAAGTACG	59.80	(TG)_9_	NOR	4	2	110	111–113
	LN829383	Tgu7, 35798065	R:	CCACCTGAGGACTAAGCCTG	59.46		SCOT	6	3		110–113
							ENG	38	2		110–112
Pha21	Osp108_F04	Gga2, 138399255	F:	[6FAM]CACAGCCTTAAAGTTCCAGCTG	59.77	(AC)_9_	NOR	4	1	146	149
	LN829384	Tgu2, 145579947	R:	TTGAGAAGCCTTCCACGACC	59.97		SCOT	6	2		147–149
							ENG	47	3		143–149
Pha22	Osp108_F05	Gga8, 19109998	F:	[HEX]CTGCAGGGAGCCGATG	60.02	[GA(CA)_4_]_5_	NOR	4	8	285	(266–452)
Unreliable	LN829385	Tgu—no hits	R:	ATTCGCCTGACCTATGTTGC	60.10		SCOT	6	3	Unreliable	(266–300)
							ENG	25	9	Poor amp.	(236–336)
Pha23	Osp108_F09	Gga2, 64794670	F:	[6FAM]GCTCAGGACAGCGAACAAAC	59.76	(CA)_9_	NOR	4	2	180	179, 183
	LN829386	Tgu—no hits	R:	CATGTAGAACTGCAGCACTCG	59.34		SCOT	6	2		179, 183
							ENG	46	2		179, 183
Pha24	Osp108_G03	Gga—no hits	F:	[6FAM]GATCTTGTTCTAACCCTCTCACAATAC	59.87	(TG)_15_	NOR	4	1	217	(220)
Unreliable	LN829387	Tgu1, 38622635	R:	TGTCATTAAACAATTCAGAAAGATTACC	60.07		SCOT	6	3	Unreliable	(214–224)
							ENG	11	3	Poor amp.	(220–224)
Pha25	Osp108_H01	Gga—no hits	F:	[HEX]CTGGGTTAAAGTCAGTGGGATTG	59.24	(GT)_9_	NOR	4	3	174	177–181
	LN829388	Tgu24, 2050527	R:	TGTCCATGCACCTATCCATCC	59.58		SCOT	6	1		179
							ENG	48	2		175–178
Pha26	Osp108_H08Z	GgaZ, 55975474	F:	[HEX]TTGAGTTGTTTTAGACTTTGACA	54.64	(TG)_9_	NOR	4	1	144	(144)
Unreliable	LN829389	TguZ, 68820524	R:	TCCTTATTTTCATCCTCACTGA	54.53		SCOT	6	2	Unreliable	(142–143)
							ENG	33	6	Poor amp.	117–141
**Pha27**	Osp34	Gga13, 10093338	F:	[6FAM]TTTAACAGCTCCCACTCTGATG	59.38	(GATA)_11_	NOR	4	5	173	164–196
	LN829390	Tgu13 4122045	R:	AGCATGCTTGTGGTGCAG	59.55		SCOT	6	6		164–192
							ENG	48	6		164–196
**Pha28**	Osp222	Gga, no hit	F:	[6FAM]GGTGGAAAACTCCCTGAGC	59.65	(CTAA)_11_	NOR	4	5	130	117–133
	LN829391	Tgu, no hit	R:	TGCTTTTGGGGTGAAAAGTC	60.09		SCOT	6	5		116–129
							ENG	48	5		117–137
**Pha29**	Osp354	Gga6, 22515994	F:	[NED]AAAGTCCAGGGCAGTTTGTC	59.19	(TATC)_12_	NOR	4	5	144	135–151
	LN829392	Tgu6, 22351865	R:	GAACGCTGTGGGACCTTC	59.18						
		Plus Unknown chr 110289344	F:	[HEX] used for UK samples			SCOT	6	3		138–148
							ENG	48	4		135–147
**Pha30**	Osp428	Gga3, 31915082	F:	[6FAM]CTCAACACAATTTCTATTGGAACAC	59.03	(TATC)_13_	NOR	4	3	247	247–255
	LN829393	Tgu3, 35243746	R:	TGGTACTAAGGCTCCATATAGGATAAC	59.35		SCOT	6	3		239–251
							ENG	48	5		231–255
Pha31	Osp537	Gga, no hit	F:	[HEX]AATTATGAGCCATTCTGCAACAG	60.50	(GA)_13_	NOR	4	1	197	197
	LN829394	Tgu9, 15738938	R:	CATCCTGTGTTGCCAGTGAG	60.31		SCOT	6	2		197–220
		And Un 58947724					ENG	48	2		197–219
Pha32	Osp742	Gga, no hit	F:	[6FAM]CTTGAGCGCCTGCCATAG	60.66	(CA)_22_	NOR	4	0	189	No amp.
Unreliable	LN829395	Tgu, no hit	R:	CACAAGCTAACAGGACCATTCTC	60.18		SCOT	6	4	Unreliable	(183–191)
							ENG	48	0		No amp.
**Pha33**	Osp1639	Gga, no hit	F:	[VIC]AGGTCAATAGGCTACGTGAACAG	59.72	GATA GATG (GATA)_12_	NOR	4	3	130	129–137
	LN829396	Tgu2, 95818547	R:	CACAGGCTACCTTAGACAACACC	60.10						
			F:	[HEX] used for UK samples			SCOT	5	3		129–137
							ENG	48	5		124–140
Pha34	Osp2311	Gga and Tgu,	F:	[6FAM]CTGGGCTTGTCCATCCAG	60.20	(CA)_11_	NOR	4	1	148	145
	LN829397	Multiple copies	R:	AGGTACGAATATACCCTGAAGCAC	59.83		SCOT	6	1		145
		in genome					ENG	48	2		145–147
**Pha35**	Osp2323	Gga, no hits	F:	[PET]GAATCCACCCTCAGCAAGTC	59.66	(G)_7_ (GT)_12_	NOR	4	2	110	103–115
	LN829398	Tgu, no hit	R:	ATAGCAGGATGCTGGAGGAG	59.41						
			F:	[HEX] used for UK samples			SCOT	6	2		109–111
							ENG	46	4		109–115
**Pha36**	Osp3963	Gga, no hits	F:	[NED]TTTCAGGTGGGCTTCATCTC	60.20	(GATA)_13_ GATG (GATA)_2_	NOR	4	5	174	166–186
	LN829399	Tgu, no hit	R:	GAATCATCCTGAAATGCTTATTTTTC	60.51						
			F:	[HEX] used for UK samples			SCOT	6	3		174–182
							ENG	48	5		166–182
**Pha37**	Osp4029	Gga, no hits	F:	[6FAM]GCTAAGTGCATCCCTTCTGC	59.98	(GT)_10_	NOR	4	3	94	86–92
	LN829400	Tgu, no hit	R:	GTGCAGCAGCCTTAGCATC	59.72		SCOT	4	2		86–88
							ENG	48	3		86–92
	Summary			Total numbers of loci polymorphic, monomorphic				L.	Poly.	Mono.	No amp./
				or failing to amplify per region samples were taken							Unreliable
							NOR	37	26	4	7
							SCOT	37	28	3	6
							ENG	37	29	2	6

^a^Loci in bold and underlined were selected for multiplexing; *ENA* European Nucleotide Archive: http://www.ebi.ac.uk/ena/data/view/LN829364-LN829400

^b^Chromosome location in the chicken (Gga) and zebra finch (Tgu) genomes (see Supplementary File)

^c^Tm, melting temperature, the PCR program used was Norwegian samples: 95 °C for 15 min, 30 cycles of [95 °C for 30 s, 57 °C for 90 s, 72 °C for 60 s] and a final extension step of 60 °C for 30 min. UK samples: 95 °C for 15 min, 35 cycles of [94 °C for 30 s, 56 °C for 90 s, 72 °C for 60 s], and a final extension of 60 °C for 30 min. Six loci were found to be unreliable in all populations, alternative primer sets could be designed if required. *Pha07* was monomorphic in the three populations tested but may be variable in other populations/subspecies.* Pha12* was homozygous in all 21 females genotyped supporting its suggested Z-linked status, *n* number of individuals tested, *Country* location where individuals were sampled, *NOR* Norway, *SCOT* Scotland, *ENG* England (see text), *A* number of alleles observed, *No amp.* no PCR amplification, *L*. number of loci tested, *Poly.* Polymorphic, *Mono*. monomorphic

^d^The expected allele size was based on the sequence of the male osprey *Pandion haliaetus* individual (02/09; that hatched at the Rutland Water Nature Reserve, Oakham, England, UK) from which the primer sets were designed (see text)

**Table 2:** Multiplex microsatellite genotyping and sexing of the osprey (*Pandion haliaetus*)

Locus and primer set	Clone name/reference	Chr.	Fluoro–label	MP set	Final primer concentration (µM)^a^	Repeat type	Pop.	*n*	Allele size range (bp)	A	*H*_O_	*H*_E_	*P*_HWE (GENEPOP)_	*F*_NULL (CERVUS)_
Pha04	Osp107_C09	3	VIC	A	0.04	Di	NOR	17	152–168	6	0.71	0.69	0.3845	−0.0350
Pha10 set 1	Osp107_F12	3	PET	A	0.2	Di	NOR	17	165–195	10	0.88	0.87	0.6130	−0.0280
Pha27	Osp0034	13	6FAM	A	0.2	Tetra	NOR	17	164–192	7	0.53	0.63	0.0783	+0.0929
Pha28	Osp0222	Unk.	6FAM	A	0.2	Tetra	NOR	17	117–133	5	0.82	0.76	0.6762	−0.0508
Pha29	Osp0354	6	NED	A	0.04	Tetra	NOR	17	135–151	5	0.76	0.73	0.1537	−0.0427
Pha35	Osp2323	Unk.	PET	A	0.2	Di	NOR	17	115–119	3	0.24	0.36	**0.0170**	**+0.2290**
Pha37	Osp4029	Unk.	6FAM	A	0.2	Di	NOR	17	86–92	3	0.59	0.63	0.3211	+0.0294
Pha02 set 1	Osp107_A08	6	PET	B	0.2	Di	NOR	17	188–212	7	0.59	0.50	1.0000	−0.1483
Pha13	Osp107_G06	12	6FAM	B	0.2	Di	NOR	17	182–196	8	0.94	0.88	0.6118	−0.0494
Pha16	Osp108_C02	4	6FAM	B	0.2	Di	NOR	17	296–302	4	0.53	0.57	0.7551	+0.0367
Pha30	Osp0428	3	6FAM	B	0.2	Tetra	NOR	17	235–255	6	0.65	0.78	**0.0323**	+0.0568
Pha33	Osp1639	2	VIC	B	0.04	Tetra	NOR	17	125–137	4	0.71	0.70	0.9226	−0.0080
Pha36	Osp3963	Unk.	NED	B	0.04	Tetra	NOR	17	166–186	6	0.71	0.77	0.3684	+0.0365
Z-002D^b^	Dawson (2007)	ZW	6FAM	B	0.2	n/a	NOR	5M	127	1	0	0	n/a	
							NOR	12F	118 and 127	2	1	1	n/a	
Z-002D^b^	Dawson (2007)	ZW	6FAM	S-plex	0.2	n/a	UK	28M	127	1	0	0	n/a	
							UK	26F	118 and 127	2	1	1	n/a	
Z-002A	Dawson (2007)	ZW	6FAM	S-plex	0.2	n/a	ENG	27M	210	1	0	0	n/a	
							ENG	21F	210 and 218	2	1	1	n/a	
Z43B	DAD et al.	ZW	6FAM	S-plex	0.2	n/a	UK	28M	272	1	0	0	n/a	
	unpublished		Ta^a^=	50 °C			UK	26F	268 and 272	2	1	1	n/a	

^a^The full PCR programs used are provided in the footnotes of Table 1. *Chr*. chromosome location (see Table 1 and Supplementary Figure), *Unk*. unknown, *MP* multiplex set, *S*-*plex* marker amplified separately in a single-plex, *Pop* population genotyped: *NOR* Norway, *ENG* England, *UK* individuals sampled in England and Scotland combined, *n* number of unrelated individuals genotyped,* M* Male,* F* Female, *A* number of different alleles observed, *H*_O_ observed heterozygosity, *H*_E_ expected heterozygosity, *P*_HWE_ probability of deviation from Hardy–Weinberg equilibrium (data in bold indicates *p* > 0.05), *F*_NULL_ estimated frequency of null alleles (data in bold indicates *F*_NULL_ > 0.2)

^b^The *Z*-*002A* and *Z*-*002D* (Dawson 2007) and *Z43B* (DAD et al. unpublished data) were used for identifying the sex of the individuals. *Ta*, PCR annealing temperature (50 °C for *Z43B* and 56/57 °C for all other markers, see Table 1 footnotes); *M* Male, *F* Female, *n/a* not applicable

## Results

Genotyping revealed that all feathers were from different individuals. The genetic sexing revealed that ~10 % of osprey chicks were incorrectly sexed in the field (5/52 errors when based only on size/morphology). Microsatellite sequences were submitted to the EMBL-EBI European Nucleotide Archive (LN829364–LN829400; Table 1; S1). Of the 37 loci tested, 31 could be assigned a location in the chicken (*Gallus gallus*) and/or zebra finch (*Taeniopygia guttata*) genome based on sequence similarity (following Dawson et al. 2006) and 2–3 were Z-linked (Table 1, Supplementary Figure). From the 26 loci polymorphic in four individuals sampled in Norway, we selected 13 for multiplex-PCR that were placed into two sets based on fragment size, genetic variation and peak interpretation in the Norwegian samples. Multiplex genotyping of 17 ospreys sampled in Norway revealed a mean of 5.7 alleles per polymorphic locus (range 3–10; genotyping was performed in duplicate; Table 2). Heterozygotes were present in both sexes for these 13 loci indicating they are autosomal. Observed heterozygosity ranged from 0.24 to 0.94 per locus (Table 2). Two loci deviated from Hardy–Weinberg equilibrium in the Norwegian population (*p* < 0.05, Genepop v4.2; Table 2); possibly due to a Wahlund effect (*Pha30*) and/or allelic dropout/null alleles (*Pha35,* estimated null allele frequency >0.2, Cervus v3.0). Despite the source of DNA being feathers there was no evidence of dropout at any other loci (Cervus). No pairwise locus combinations displayed significant linkage disequilibrium (*p* < 0.01, Genepop). The combined probability of identity for the 13 loci was 8.0 × 10^−12^ (GenAlEx v6.501).

In conclusion, this multiplex set of novel microsatellite loci combined with the sex markers will be useful for genetic analyses of osprey, including typing non-invasive samples, such as shed feathers.

## Electronic supplementary material

Supplementary material 1 (DOC 154 kb)

Supplementary material 2 (DOC 57 kb)
