# Supplementary material for: A multiplex microsatellite set for non-invasive genotyping and sexing of the osprey (Pandion haliaetus)
Source: Conserv Genet Resour. 2015 Oct 29;7(4):887–94. doi: 10.1007/s12686-015-0497-4 (PMC4657739; doi:10.1007/s12686-015-0497-4)
Supplement: Supplementary file 1 — Supplementary material 1 (DOC 154 kb) [file 12686_2015_497_MOESM1_ESM.doc]

**Dawson DA,** Kleven O, dos Remedios N, Horsburgh GJ, Kroglund RT, Santos T, Hewitt CRA (2015) A multiplex microsatellite set for non-invasive genotyping and sexing of the osprey (*Pandion haliaetus*). *Conservation Genetics Resources*.

**Supplementary Table S1** One hundred and seven osprey (*Pandion haliaetus*) microsatellite sequences.

| **Locus** | **Clone name & ENA sequence accession no.** | **Sequence** |
| --- | --- | --- |
| Pha01 | Osp107_A01WZ, LN829364 | GATCTGGAAAGGCTAGAGAGCTGGGCAGTCACTGACTGTATGAAATTTAACAAGAGCAAGTGCTAGATTCTCCACCTGGGATTGGATAATCCTGGTTATACATACAAACTGGGGGATGAGAGGCTGGAGAGCAGCCCAGCAGAAAGAGATTTGGGGGTTTGGGTTGATGGCAAGCTGAATATGAGTCAACAGTGTGCCCTAGCAGCCAAAAGGGCCAACTGTGTCCTGGGTGCATCAAGCACAGCATGGCTAGCCGAGAAGAGAGTGAGAGTATGTGTGTGTGTGTGTGTGTGTATACGTGTGTGAGGTGTGGGAGGAACAAAGCGGCCATGCAATCAACTTTGTATAAAATTGGCGCCGTGTCCAAGCTTCCCGGGTACCGC |
| Pha02 | Osp107_A08, LN829365 | GATCACCAGAACTACTTAAATACCTTTTTGAAATAACTAAAACAGAGGGACACAGAAAACAGATATTACAAAAGGAAGAATAAAATTTGCATCTGTTACGTGAGATACTGTGTTCAGTCATGTGCCTGCTCTTTAACCATGTTATTTCCACATGCGCATTGAGCAGATAGTGTAAACCTCTTGGTGTACCAAACCAAATCTTTGTCGCTCAAAATAACAAAAGTTTGTTAGCCTGACAGTCCTGGTAGAAGACTGATTATCTGCAAGGCCTGGTGGAAAACTGTGACCTACTAAGCCCTCTTCAATTTACTTTACTATAATTTGTTAACGTTCACTCACACACACACACACACACACACACACACAAAAGCATTAAAAAAAAGAAATAGAAGTCAGAGTGACTTTGGATTTGCTTGTTTCTAATCTGCAGCACTCTACTACCTCCTCCACTCCTGTGATTTGCCATCAGCACATCCTCCTCAGGACATGAAAGAGCATTTCCAAGCAGCAGACACTGGCTTTGATC |
| Pha03 | Osp107_C03, LN829366 | GATCTTGAAGACCATCGCCACAGTGGTGACCTTTGTATGCTGGCGTGGAGGCTGCAGTGTGCCAAGAAGCTCAGTGTGGAAATTGGACTGAGAGGAATGGCTGTGCCTCAGTTTCTCTGAGTGCAGGATGGGAGAGGTGGGGCAGGAAAGGCAACTGTCCACCAACCTTTGCTGTGTACGAGAGGTCTTACCGTTTTCTGCAGAGATTATGCCATATTAAGATTACTTTTAAACCAAACCAGTTATTTTCAAGGTCCTTTTCCCCCATTTTATTCCCATGTAACACACCTCTGTAGAGCTCCTGTACCTTACTGGCAAATCTAGCCCATCTCCAGTGAATCACCTGTAGTTTGTGTGTGTGTGTGTGTGCGTGTGCGCGCGTGTGCGCTGGGCACGGTGGAGTGGGGTGGGACTGCACCAACTTTCTAATTTCATTAGGCCCAGTAAAGCGGGCGCTTTGGATC |
| Pha04 | Osp107_C09, LN829367 | GATCTGTATTAACAACGACAGGCTAGTCCTGCTATCAACCTTTCATCTGAAACAGCAGATGTTTGTATGGCTGATGTGAAGGTTATAACCCTACTGATGACCAGTCTGATGCCTTGGGATAAATTCTCTCTTTCTTAGTTTGCCTTCATAAAAACCATGGCAACCACACACACACACACACACACACACCACCAAGCAAATAAACAAATATACCTATGTGAAAAGAAAGTGAAGTTGCAAGAAACCCTCCAAATGTGCAAAAGCTAGAAAATGTCAGCATTAATTTGTTAGTACAACTTTGGTGTGCTCCACCACACGCATGCTTAATATTACAGCTTATGGGATC |
| Pha05 | Osp107_C10, LN829368 | GATCAAACAAAACTGTCAAAGTGAAATCTTGGCCACTGAAATAAAAAATAAAATTCCTTCTGATTTTAGTAGCACCTAGATTTCATTTAACGGTTTAGAAAGTGAAGGCACTGAATACAGCCCAGTGTGAAATTACCATTATACTCTTAATTTAGCATGCAATGGAATTGTATGGCATTCAGAGAGTCATTATAATAAACATCATAGTAAAGTAAACATACATTGTCCTAGATATACACGTGTGTGTGTGTGTGTGTGTGTGTACACATATAGTGAGAACATAGTGCGTGTGAGAGTACTTCTCTCATATGTGCCGCTTCATTTGTTATTCATTTCACTACAGATC |
| Pha06 | Osp107_D06, LN829369 | GATCCATGTACAAATAAATGTATGATTCTTGTTGTCCGTACTCAAGCTTAAAAGCCTTTGGTTTATACTTCAGGGGGAGTTCATTTCATTTTTACACGTTTTTAATTGTTGCACCTCACCAGTTCTAGGTGGCACACAGGCACTGATGCAGAGCTTTGAATTAGTGAAGATTTCATAATGCTACCTGTTCCAGTCAAGCTTGTAGCAGTCTTGCAGAAAAAATCACACACACACACACACACACACACACACACACACACACGAGTTGTATTTATGGTGGATGAATCTGCCCTGCTGCTTCTGTACAGGCAGATC |
| Pha07 | Osp107_D07, LN829370 | GATCACCTCGCTCATCTAGGGACACCACAAATACACACACACACACACACAATCCTACTTTCCTGTCTGTTCAGGATTCCCAGCAGCACAGTCCCGAACATTTCAGAGGCTCTCTTTAGTTACGTTACTGGGCCTGTGACGCTAGAACAAGGAAATATTCACCTGGAAAGTGTCTTCTTCTACATGAATGAGTCTCATTGTCAAAACCTTGAGCATATATAGGTGCGTATATGTTTTGCAGTGGAGAATTTAGTTTCTCGTTTATACTGTAAAGCTAAAAATTCTGGTAGCTTCAGGCTTGTTTTTACAAGCTCCCAGCTGTACAAGGATTTCTGTTTTTACAGGAAAAACTAAGCAGTCCATAGAGCATGATGGCTGTACATCCATAATGTGAGCTTCATGAGGAACAGAGCAACTTTATTATGATC |
| Pha08 | Osp107_D08, LN829371 | GATCTAAACCCTAAAGTGAAGCCATGACGAATGGTTAGGAATTGTGCTTGTAGGTATTTTTCCACAACATTCTAGCCTATGCATAAAAAAAAAACAACGTTAAAGAGAATGAGTAACCACATAAGTAGAAAGAGTCATACAGGGAGGTCAGCCAATCACCACATATAGGTCTTTGGGTTGGGTTTCTTTTCCAAGTAAACACACACACACACACACACACACAGTTAATTTCTGTCTCCAAGCTCCCAGCCAGGAAAATACCTGCCACAATAATACCATGCAGGAAATCCTGCAAACTTGATGCACATTTTTATTAGTTTATCTTGATACCCATGTAGGCAAACCCTAAAATAAAAGCAGAGCCAAGGTAGTAGAGGTTATGTTATTCTGCAAGATC |
| Pha09 | Osp107_F09, LN829372 | GATCTACTAGAATGGCTATTGCAAATATGCCTGTGGGGAGCAGGTGTCACCGGCGGCAGGGGAGAAAGAGCAAGGGGGTGGTAAAGAATTGTGCAGAGAGCAAAACTCACTTGCAAAGTCTAAGATTTCTAGTGATAACCCAGAGATGTTCAGTTAAAAACACAGGTATTGTGCTTGCTGCCAGTTGCTAGGAAATCATATAAATATTTAGGATATATAGCATACACTGTAGTTTGTAAATGATGAAGCACACACGTGTGCATATGTGTGTGTGTGTGTGTGTGTGCCTGTGCATGTGTAAACTATCCCAGACCTACTGCTGAGGAGCTTAAAAACAAAGGAAGCATGAGTGACTTTAACTATCACGACATCTGGTGTAGACAAATACAGCTGAATGTGCCTCATCAACTGCCTTCCCTAAATAATACAGAGGACAAATTCATGATC |
| Pha10 | Osp107_F12, LN829373 | GATCAACACATTTCCTTTTGGAATATGTCTTTTTTTCCCCTGGTGAGAAGCCCAGTGAAAGTAAGATAGGCACACACTCATGCACGTGTGTGTGTGTGTGTGTGTGTGTGTGTGTGTGTGTGTGTGTGTGCATGCACAGGTATGTGTGTGACAGAGAGAGAGACTTCAGGGTATTGTGTTCAGAAGTTAATTTTTTTGAACAAGGTGAAGGGTAATGTACACTTTTTATACCTTACTAGTATTATGTATTTGTGTTATATATAACAACGGCTAAATGCTGTGACTGCCACTGCAAAATAAATTCCCTGTAGATTTCATTGAAAAGCTTGTGCCACCTTCACTGACCCATTCAGAAAGCCCTCTAAAAGGGTACAATAAATTGACATGGCTAGACACTAGGGTGATAGATC |
| Pha11 | Osp107_G04, LN829374 | GATCATTGTCTCCGTTGAAATACTCTTTAAGATTTTCCGAGAATGAACATTCTTTTCTGTGACTGAAGTTTTAAACAACCAAATCCATACATATACATGTAGTTTGTGTGTGTGTATATATAGAGATATATATATAATGTGTGTGTGTGTGTGTGTGTGTGTATATACACGGTATCTACATATGGGTGTATGTATGTGTATACTCATATAACCTGTATGTTTAACTGCATTTAAAATTAAGTCAGAAAAATACAGAGTTAAATAACATATTGGAGTAGTAGCAAAATAAGTGTTATACTTCTGTTGCTAAGTGTAGGTGGACAGATGTTACTGAGAGAACAATTAATACTCAGCTCATGTCCTTAAGCCAGAAAGAGTTATACTTAATTCCTGAGCAGTTATATGGTAGAAATTAACCCAGAATTAAAAACTCCAGGAAAACAGAAAGCATTTGGCTAGTCTTAATAGAACAAGAACTCTATGATGTTATCCAAAAAAACCCTTGGAGAATTAGTCCAGGACACAAAGCTGTGTGCAGCAGTCCAAAACCACAGGGAAAGGAGACAATATTACTCAAGAGTTAGCTAAACACCAATTGGGACTGACTGG |
| Pha12 | Osp107_G05Z, LN829375 | GATCCAAAGAAATAGGTGGGCATTTTAATACGTCAGAGGAAAATTACAAAAAACTTTTGACCAGGTTGATTGACTCATCACAGCAAACATCTTAAATAGTTCTTTATCAGAGTCATACAGAAACCTCAGGGTCATATATTTTCCTTTTCTTGCATCCTAATGAACCTTTGCGCACGCGTGCACACACACACACACACACACACACACACAGACTAGTGATTTCACTCTTATTCTTGTGTAATTGATAGCGGTACACCTTTGAAGAAAGTTTTAGTTTTGTCTAACAGATGCTTTAAACAGTAGGAAAAATGTTTGTTAGAAAATTCCCAGACATTTCTAGCCCATTTGTATACATCAGAAAACAGTGGCTTTTTAACCTGGTATATTTGGGTTTTAGTATTCTTGCCTTGTCCTTTTAGCCGGCTGCATCCATGTTTAACCACCAGCCTCTGGGCTGCAGATAGTATGGGGGAGAAGGTACTGCTATACGTGCAGCTGATC |
| Pha13 | Osp107_G06, LN829376 | GATCCACATCATGACTGATGAGGGAAGTGAAAATAAAGGTAAATATGCCACTTGTATCCTGTTTATATTGTAGTAGATGAGACAAATTACTTTCTGCCCTGCTTACTATAACCAAACAAATGGCGTTAAAAATATGTGCGCGCGCGCGCACACACATACACACACACACACACACGAGTTTAATCCATAGAGGTATGGGGGTCTTTATCAAGGAAGCCAATTTTAAGTAATTATATTTAAAGAGTAATACAGGGAAGTCATGTGCAGCTATGGAGCAGAAAGCCTCAGTGTGTTTTGTAACTCTGTAATAACTATTATTTATCACAAATGTGCTGTCTGTTTGTTCACACTGAGTAATGAATGACCTTTCTGTTTCCACAGCTAGACCTGTGCAGACTTCAGCTGATGGGGGTATAGTGCAGTAGCTATTTATCATGCTTCACTTACACTGATC |
| Pha14 | Osp107_G07, LN829377 | GATCATTTTTCCATTGAAAATTTGCACGTTATTTTCATTATGTTCTTTAAAATCTCATTTCTGTCTTTCTTGGTATCTCTACTAAATCCTGCTGCTAATTTTAAAAATAGCGCTTCTTTCTTGAAGCAGTCATTTACACATTGACACAGTGAATCAGTTTATATTCCTTGATGTTTAGAGATGAAGCCATACTTACATGAAAATGGAGAGTGCATTAGATATTGAGAACTAATGCTGAGCCCTACAGGTCAAGGGCATTCGGAAAGAAGACTCTGGCTTGCAGCTCCCTCTAGCACACACACACACACACACACACACACACGGGCAAAAGATAATGAGTCTCCACTCCCATTTAATAGATTAATAGCTTTTAGTGCCAGAAGCTTATACTTTGATC |
| Pha15 | Osp107_H11, LN829378 | GATCTCTCAAGCTACCTGTGTGCTTCTCTTCTCCACTGAATGCCCTGAAGAGGGAGTCTGTTGTTTTCCCTAGTTTTGTGGGGAGTGTGACTGATGGCGGTCAGATAGATTGACACCACTCATTGCTTGCAGAAGTGTCCTCCATGGGGAATAGCTTACAGGAGAACTGGGCTTGGTCATTCTTTTCTGGTGAAATTGACAAATCCCTGTGAATGTAAGTTTGGAATATATATATATGTGTGTGTGTGTGTGTGTGTGTATATGCATATATATATTTCAACAAAGGAGTTGGGTTCAGAGTGACAAAGATGCAGGAAAGGCAGGTAAGTCTTTCCTAGAAAATGAAATAGGGGATTTTCAGTACTGTCTTTGCTGAAATACTGAAAGGGCTGTGTGCCATGGGTGGGAGCCCTCTTAATTCTTTTGCCTCCAGTGGGATAAGACTGAGGGCTTAGCCCTGAAAATCTGAACCTTTTCTAAAGGTGCATTTGCTTTTATGTGATTTTCTTGGCATTCAGCACAGCCTGAGAACATATTCTGCTCCATGAAGAGGATC |
| Pha16 | Osp108_C02, LN829379 | AGGCATTCCATCTCATGCACAGCACTAAGTATATATTCCCCACCAGCAGTGCCACACAGCTCAAAGACCTAAACAGACATGCTATTAGGCCATTTACACAATGGTTTTGTTAACACTTTCAGATGCATGGCTGAAAAGTACATTAAGTACAAGCAGTACATTTTCTTTATCATTGAGCAAATACAGACCAATATACGTGCCTTAGCTCACTTAGATTTGTGTTCAAAGTGTTGCATAGCTCTGTTATGCCCTAATCCATCAGAGTTACCTGCCGCTGAAGCAGCAATTACAATTCAAGTTCCAAAATGTTCTTGCACTGAAAGTCACTCACAGTTAACATTGCAGCTAAAGAGGTGCCTGCGTATTTCTCTGTTACTGTGTTTTAGGACATGAAAGACCATCTAGCTATTAGAGAAAAAATAAATCCTGAAACATATACAGTTACCTTATTTTTGTTTTGTTTCTGTATGAACATCTGATATGCTAGCAGTCAACATCTTGGAAGAGGTTTCTGTCCTTGTCAGTCTCACTGGAAATGAAACACAGTGAGAAGGACAGATGAGTGTGTGTGTGTGTGTGTGTGTCTGCATGTATGTTAGAAGAAAGCTGGGGGGGTAATGTGTAGCAAAGGGGTGGAAAGGGAAAGGCCACGGCTTGTCTCCTATTCCTTGATTCGAGCCTTGCTCATCCAATGGATC |
| Pha17 | Osp108_D06, LN829380 | GATCATTTGAGTCAGGGTTGTAGAATAGAGGAAAGGAGGTAAAAAAGGAGGACATGGTAACACTTTCAATGACACAAAAATCTGCTGGAATGAAGAAGGAAATAACATTGGTTTTCATGTCCACAGTGGACAGGGACAAGAAAAGACTGTTGCTTAGACAAAAGACACAGGATACAGGAAAGGCAGGTGTGTGTGTGTGTGTGTGTGTGTGATTACAAAATAAATCAGCTTTCAATGACAGAGATGATGAACTACTATCACACATTGCCTGGGGAAGTCCCAGAGTCACTGTCTTTGGCAGTTTCTAAGAAAAGGATAGTCATTCATAGCATTAAGAATGGAAAGTCCATAGAGAGAAAATATTCCCATGCTGGACTAGATGACCCCATGAAGTTCCTTGCAGTCCTGTGCCTCTACGATTCTGGCAACTTATACAGCACATATTACACTATCAATCTAGTCATGTATTTCTCACAAAATACTCATGGAAAGATGTACCCATAGACACCTACAGATTTCAAACAAAGAAAAAAGATC |
| Pha18 | Osp108_D09, LN829381 | ACTTCCCAGGGAACTGAGGTGAGGGAAGTGACAGCAGGGCTATGTGTGTGTTGGGTGCAGATGCAGCACATAGAGGGGGTGGCTGACCATCCTTCCTGAAGGAAGCAGTGTCCAGGACAAGAGGGCAGGGGAGACCTGGTGGCTTGAGTTCGGACCTCTGGCTGATGCACAGAGGAATGCTGCTATGGGCAGTGGTATTCTTTGCACCACAGAGAAAATTGCAAAGCCGTGCAAACCTCTCAGAGCTAATTATTTTCAACTACTTGAGTCAGTTCCCCTGCAGCTGCCTGTGTCTGGTAATAACAGCATCTCATACCATGCTATTTTGGAGGAGTCAGTGATTTTGGAGTTTGACTTTGCTTCTAAAAGCATAAGAACTTTGGTCACTTCTGTGGAACCTCAAACGTGATGATACTAACCTCTCTCTCTCTCTCTCTCTCTCTCTCCCCCAACCCCCACCCCCCCCACCCCCATGTCCTTTTATGCAGAAGTGGGTTGGTGCATCCCATAAGAAAATGACCCCTGATTTCTTTGAATGGGGAATAGTCCTTCCAGAAGTCCTAGAAGTTTACACCATGCGTCCTTCCAACAGCACCTCACGGGAGGAGCTGCATGAGCTGAGAAAAGATC |
| Pha19 | Osp108_E06, LN829382 | GATCTATTACTCATGGTGTCGTGGTGACTGCAGTTTAGTTCTTGCAGTCACTGGGCCTTTTAGTGTGTGTGTGTGTGTGTGTGTAAGAGCATGGAGTGAATCGCTTCAGGAAAACACTCCCAGATGGGAGGCCCTTAATCCAAGGTCAAAGGGAGGGCTGGAGGGAGGAAGAAGCCACCTTTTCAGCTTGTGTGAGTCAGCATAGGTCTAGTAGTTCTCTTCCCTGCAGAAGATGACAAAATACACTGTGCTTTTGTGTTGCATAAATAACAAGAGGTATAAGGTTGTGTTTAAAAAAAAAATTGTAGTGCAAAAATGTTTTTCTCCTCATCCCTATGTATTTTAACTGCTTTCAATGTGACCTTGTTGATGCTAGGGTTCCTCATGCTGTTCAGCTAGCACGTAGGCATCTGCCTCAGGGCTGCCAGTGGAGAAAGACACATTTAGAGTGACTGCTCACTTGGGAAAAGTTCTTGATGGGCTTCCACCATGGCAGGTCATGACCTATTTGCATTGTCAATAGAAGCGCTACTTGTCCTATCCTCTTGGGATGAAGGCTAATTTTCCCCAGAGAATGAAGGATC |
| Pha20 | Osp108_F01, LN829383 | GATCCTTTACCTACAAAAGCCAGTTGAGGGTTTGTGTTTTGGTTTTTTTTTCCTTTGGATTGGAGAAAGCTTTATTTCTGTGACTGCAGATATTTTAAAATATATTTTAAACGTTTTCTATTATTCTCTCCTTCTGCTCTCCACACATGTTAAAATCCGTTCATAGCAAGGGTCTGCGAAAGGGGCTGGAAACAGCTATGTCCTTTTTCCAGGGCTTGTTTTCTTTGTGAGCCTGCAAGTACGTTGCATTTGTGTGCATGTGTGTGTGTGTGTGTGCGTGCATGTGCGTGTGTGTGTTTGTGTGTACGTGCACAGGCTTAGTCCTCAGGTGGAACTGTGGTTCCTGTGCTTTCGTTCTCGAAGCTGTTATTCTGAAGGGAATGGAGGCAGCTGTGTTTACAGCTCAAGCCTTGGCATACATTTACCAGAATCTATTATTCATATCGTTCAGTATCAGGGAGAGATC |
| Pha21 | Osp108_F04, LN829384 | GATCACAATTTTACTGTATTGAGACAGTTAGCATGAAGTAAATTCAGTTGACAATTTAATCATGAAATGAGAGGCACAAGTCCTGAAAAGCCCTGAAATAAGGGGCTTTGGCACTGTCACAAGCTTGCTATATTAATGTGTTTCTTTGAAAAATTGCTTGGGTGCTCTTTAATTTACAATCTCACAGCCTTAAAGTTCCAGCTGTTCAGCAGACTACTGAACACTGTACACAGTGATTTGCATTGTATGTCACTGAACTTTTTAACACACACACACACACACGCACACTCTCTCTCTATTTTCTAGCAGGTCGTGGAAGGCTTCTCAAAAACTTTTGCTGTCATCTAACCTAAGATC |
| Pha22 | Osp108_F05, LN829385 | GATCAGAGTCTTTTCGCAGCGGGCGGCTGCAGGGAGCCGATGGGTGCCCCTGGGTGCCCCTTCAGACTCCACACACAGACAGACACACACAGACAGACAGACACACACAGACACACACAGACAGACAGACACACACAGACACACACAGACACACACAGACACACACAGACACACACACACAGACAGACACACACACACAGACAGACACACACACACAGACAGACACACACACACAGACAGACACACACACGCCCTCAGGTGCCTCCTCTGTTTCCCGAAGCGGAGTCTGACGCAACATAGGTCAGGCGAATTTATTGAGTGGTGCAGCGGTACTGGACAGCGTGAGCTGGGTGCCTCCAGACAAGGACCCCAAAGAAAGAAATCTCTGGGCAATTATACCCTTACAATCTAAGTTCCCCTCCCCTCACACAACGGTTTGTCCAATAGTAATATCTTGGGTCTGGGGTCTTCTGGATC |
| Pha23 | Osp108_F09, LN829386 | GATCAGGACTGCAATGATTAGGTTCTGCCACTCCTCCTTACCGTTTCTCTTGCACATCTTTTTCTAACAGTATTTCAGGGCTCTCCATCCTAAGGGTAATCACTGTAGGCTACGCACACAGGCCAAGACCTAACATACTGTTACTTGCAAAACATTAGAAAGCGAGCCAGAAAGGCTGCCATTACGAAGTCTGAACACCCCCAAAAAATTAAAATGCATTATCCCAGCGCCCCCCCATTGCTATATACGAACATATAATTCACACAAGCTCATTCCATCCTACTTATACAACTTTAATTTTACTATATGCAATCTGTATGTGCAGTACTGTTCAGCTCCAATTATACTGCAACCCTGTTAAAGTTGGCTCAGGACAGCGAACAAACACACTGTGCTGTGAGGAAAATCCCTGCAGCTGTGCTGACAGTTTAGACGGTGTTTAATCCCCAGGGTATTTCTGTGAAATTATCAACCTACTACTTTTTTTTTTGCACACACACACACACACACCCACACCCCCCAAAACGAGTGCTGCAGTTCTACATGATGCACTTGATTGTTTCAAAGCCTGCTTTCCAGACTGCCTGCTCTTCGAATCACCAAATTAGCAGAAGATAGAGTAATTACTGGTTCACCTTGAAGCAAAGATC |
| Pha24 | Osp108_G03, LN829387 | GATCTTGTTCTAACCCTCTCACAATACTCTCCCCAAATGGTGTAAACATTTTTGTGCTAATTAAAGTATTTTTACTTTTATAGGTATATATGTAGCTATATTTTATACATATGTGTGTGTGTGTGTGTGTGTGTGTGTGTGTACGTATGCGCAAAGGAGTTGTCTGAAAAAGCATTGGAAAAACTGCCAGGTAATCTTTCTGAATTGTTTAATGACAGAAAAGCAATGTATAATACTTAGCTCTTTCTTTAAGGTGTCTATAAACTGTTCTTTCTTTTAGACATCCATGGAGAAACTGAGGAGCAGATACATATGTTAAAGTGCTAAGTGGGAAATTGTGCACTCTCCCTTTATTTATTTATTACTTTGGAATTTTTTTTAACTTCAGAATAATAAAGCTGCTTGTCTGGTGACAGGAAATTCCAACCTAGTAACTGACCAGGAAACACTCTGTTCAAAATTGTGATC |
| Pha25 | Osp108_H01, LN829388 | GTTCTGTGCATGATACGGTAAAAAGGTAAGACTGCTGGAGACCAGCTCCATCCTTTGGAGATTTTAAAGATTGTTGCTGAGCAGAAGGGCAGTATAGCCAGGTTTATCAAGTTGAGGATTATCTTGGTTCTGGGTTAAAGTCAGTGGGATTGAAGGAGAGTCTGAGAAGGGCCAGTGTGTGTGTGTGTGTGTGCATGTCTTTGGGATGGTCTGTGCTGTGTTAGCCCTAGAGTTTGACTCTCAATTCCCTCCCCAGGGATGGGGTTGTGCAGCTTGGAAAGGGGATGGATAGGTGCATGGACATCCAGGAACAATTACATGGGGCATTTTATCTTGCTTGATAAGAGCCATGTTCAGCAGTTTTTCCCCTGGCAGCATTAAAGGAAGGGAGCACTTGAAGAAGCTTTTTCTAATTTTCTGTTTCCTTTCCAGCAAAAGGGAATGAACATGAGCCCACCAACTCCTTGTTGGTCAAAACAGCCCTTTCATAAAACACTAAATGTGTCCGGAGCTTCAGAATGTGCTATTCCCCTAGCAGTTTGTTAGTATTTAAGCACATTGTAGTCAGATATTTGAGTCTAATTTCTCATTTGAATTTCTTTGGTTCATCCTGCAGTCATTGGGTTGTTCTTACCTCTTTTTTTGGCAGATTAATGGGGCCTTTAGTACCTGCTATTGTCTCCCCACAAAGGTTTTTATCTGTTCTAGTCAAGTCACTTCTCAATCTCCTTTTGATAAACTTAAACATATTGAGCTCTTTATGTCACCCTCCAGGGAGCAATGGATC |
| Pha26 | Osp108_H08, LN829389 | GATCTCAATTTTCTTTGAGTTGTTTTAGACTTTGACATTATAAAAAGATGTATTTGGAAACAGATAAACATATAAATATTTGCAATTACTTTTTGTGTGTGTGTGTGTGTGCTAGTTTTAAATACCTGAACAAATTTCAGTGAGGATGAAAATAAGGAGAAAAATGCAGACACCCATTTATTCTCATTCAAATTTTTCTTCAACTAACTTAATGCAATAACAGAGGAATAATTTTATTTGAAGTTTAAATACCATTCAGCTGAATAAAGGGATGGTTATTTAAGGAACAGATGCTCATAGGTGTTGTTTGCTACCTTAAGCTATTGATGAAATCACTTGATTACACTACGTTGTAGAAGTCACGTGTCTTCATAGTTTTGAATTACCACCTGAAAGAATCCAAGATTGATC |
| Pha27 | Osp34, LN829390 | TAAAGAAAAACTGTCATATTTTTTTCCTTCTAATGGCCCTTCTGTTGAAAAAAAAAAATTAAAAACTGAAAGTAACTTCCACGGGGATTACAGCCTCCACCACTGGGGAGGCCAAATGCAATTATGGAGCCAATATTTTTTAACAGCTCCCACTCTGATGGCCAATAAGCAGCTTGGTATTCCCCCGTCTCCATCCCCCAAGCAAATTGAGATAGATAGATAGATAGATAGATAGATAGATAGATAGATAGATATATCTCTCCACTGGCAGGCTGCCAGAGTCTTTGGAGGCTCTGCACCACAAGCATGCTCAGTGAATCCTGGCCATCTGGTTAGCAGGCTGGATCCCAMRMATCCCGGGGACCGCA |
| Pha28 | Osp222, LN829391 | GCACCGAGGGCCGGGGCCACAGGGGGATCTGTCTGTGGTGGAAAACTCCCTGAGCAGAATCTCTCCTTTCCTTTAAAACTAACTAACTAACTAACTAACTAACTAACTAACTAACTAACTAACCCCTCAGAACTTACTTTTCCTATGACTTTTCACCCCAAAAGCAGAATCTGCAACTTTAAATGGTGATTACTTCCTTATGAAGGGCTGAGAGGTTTTGTCCATGTAATAAATGTGACTGCAATTGTTAGGGTTTCTTCATCTTTGCACAGACCCCAAGCTCCCG |
| Pha29 | Osp354, LN829392 | CCCACTGGTGAAGGCTTAAACCAAAAGGGGTGAAAAGGCTCATGACTGGCATCGGTAGTGGAAATAAAACATCTTTATGTGTCATCTCTAGTGGAAAAATCTGGCATCTCTAGTAGAAAAAGTCCAGGGCAGTTTGTCTGGAGAAGAAAAGTCAGAAAGTGCTGCTCTGTCTAAATTTCTATCTATCTATCTATCTATCTATCTATCTATCTATCTATCTATCTATCTGTCTATCTATCTCCATGAAGGTCCCACAGCGTTCGCATCGCTGTCCCGCCAGAGGCGATGGAGGAGCAGGAGGGAGTGGGTGCAATACAGACCTGGTAACATTAGAGGGAGCTTTGGGAAGGGGTCTGGGACGAGTCGCAAACAATAACTAGATGTAAATTACATTTTGTGAGTTTGTTTAAATAAG |
| Pha30 | Osp428, LN829393 | ATCAGTGACGGCCACATTTTTAACATAAACACAAATGTAAATGTTCTACCATTTTCCCCTCAACACAATTTCTATTGGAACACAAAATTTATGCAGGCAGACTTACTGTAAAATAGTACCTGACAAACACAGTGTTTCAATTCTATTTGGAAAATATTTGTTTTCCAAAGTATGAACTTATCTATCTATCTATCTATCTATCTATCTATCTATCTATCTATCTATCTATCTCCTTTTACAATTATTAATTACTTTGGCAATCTTATGTATAGTTGATAGTTATCCTATATGGAGCCTTAGTACCAAATGTTAAAAATGCACAAAGGTTTCAGCTATAAGTCCTCTTTTCCTAGACTAATAAAGCATTAAGACTTATTCATCTGTGTAGATCCCAAGCTTCCCGGGT |
| Pha31 | Osp537, LN829394 | AAAGTTAAACAACCACAATGTGTAAGTGCTTTTGAAAATTATGAGCCATTCTGCAACAGAAAGTCCCTTTTCTTTCTTCCTTTCTTAGCTGTCCTATCTATTTATATTATAAAAGTGGTTGGAGGGGGTGGGGGGAGAGAGAGAGAGAGAGAGAGAGAGACTCCATCCTCGTGTGCAGCATCCAGTGGAGCTGCTTCCATGGGGGAATCACAGCTCACTGGCAACACAGGATGAATATGGTGAGACTTGGGAGAAGTATAAAACCATGTTGMCAATGCCA |
| Pha32 | Osp742, LN829395 | CCCCGAGTAGCTTGAGCGCCTGCCATAGTGCTGATTGAAGCACAACATTTTCAATCTTTACACACACACACACACACACACACACACACACACACACACACATATCCTTACTCCCGTAAGCAGCGCTGTGGGAATCTCACTGGGAATGGTGTGTTTGTAAACGGCCTGCTGCAATTGAGAATGGTCCTGTTAGCTTGTGTGGGGGATTTTGAAAATATATAGAAGGTGTGTGTGGGTGTGTGTGTTTTGTTTTTAAGCACTGCAACTCTAGGATATATTCCAATCTTTACTTAGGCAG |
| Pha33 | Osp1639, LN829396 | CTTTTGAATCAACTCTGGTTTTCATCTCAAAATTCCCAGGGCCACATGCCTGCTTTATTTTCTCTTGAAATCAGAGCTGGCTCGAGTGCCTAACTACAGGTCAATAGGCTACGTGAACAGTTACAGGTCTGATAGATGGATAGATAGATAGATAGATAGATAGATAGATAGATAGATAGATAGATAGATGACTTCTATATTTAAGGTGTTGTCTAAGGTAGCCTGTGTCTTCAAAAGACATAGCAAAGCAAGTATCAGGTTGTAATTCCTCCTGCTG |
| Pha34 | Osp2311, LN829397 | ATTCAATCCCCTCGTCCAGGTCACTGATAAAGATATTAAAGAGAACTGACCCCAGTACTGAGCCCTAGGGAACACCACTTGTGACCTGCCACCAACTGGATTTAATTCCATTCACCACAACCCTCTGGGCTTGTCCATCCAGCCATTTTTTTACCCAGCATTCCTCTGTTCTCTCCCAAACTCAGATGTATATGCACACACACACACACACACACACGTGTGCACACACCTTACAGGCAAAATGTCATGTGCTTCAGGGTATATTCGTACCTCATTTGAGATGTAGAGAAGCAAAACAGTGGAGCATTCCCATGAAAAACAGTGTTGCCTACAGGAAGTCTCTCAATGCCACGAACAGCACACAGGGCCTGTCGTGGCACATCAGCCTTCATCCACTGCTCAGTCTGTATTCGGACAGTGAAGCAAAGCAGTAGCGTATGATCTT |
| Pha35 | Osp2323, LN829398 | AGGTCCGGCTGGAGGCTAGTCACTAGTGTRAGCTAGTGGGTCAATACTGGGGCCAGTACTGTTTAACCACTTCATTATGACCCAGATGATGGGGCAGAATCCACCCTCAGCAAGTCTGCAGATGATACAAAACTGGGGGGGGTGTGTGTGTGTGTGTGTGTGTGTGGCTGATTACGCCCCTTAATTCTCCTCCAGCATCCTGCTATTTTCATTTCAGGAGGGGGCTGGTCTGCTTTAGGCAGCCCTCAGCAATCCCTCTTCCAAGAATCTCCCAAGCTTCTTTCGAACTCATGTAAATAATTTGGACCCACAGCAACCTTCAGCAAGGAGTTCCTCTGATCCCAAGCTTCCCGGGTACCGCCCAAGCTTC |
| Pha36 | Osp3963, LN829399 | TTTTCTGCTGTTGTGAGGCGGCTTGCCGGCCCAAACTTGGACTCTAGATAATTATCAGCCTCCTCAACACTCCACTCCCCTTCTTTACACAGATCAGCTAGTTCCAAGCTGTAAATATTTCAGGTGGGCTTCATCTCACCTTATTTTAATCACCTCGAGTGTAGATAGATAGATAGATAGATAGATAGATAGATAGATAGATAGATAGATAGATAGATGGATAGATACATATCTGAGCTAGTTAATTTAGGGGAGTGTTAGAGAAGAAAAATAAGCATTTCAGGATGATTCATCTGACCTATTTTAGTTGATCTCATTTAACACAT |
| Pha37 | Osp4029, LN829400 | CTTCAATMAAGAAGGTAGCGAGCTTCTGGGTAGAGCTAAGTGCATCCCTTCTGCTGCTTGCAGGGAGTTTTGTGTGTGTGTGTGTGTGTGTCTCCTCTGACAGAGGAAAGATGCTAAGGCTGCTGCACCAACGCTGGACCCAGGGCTGGTGAAATCTTTCTCTACTCTGCCTCTGTCTCTTTTTTCCCCGTAATCCCTATACCTCATCCCTTTAGACATAAGCCGTT |
| Pha38 | Osp107_A05 | GTTATGGGAAGATTACTATCGCTTTGAACCCTGGTCCGCTCTGCTCTTGGCTGGCGTCAGGGCCACAGAAGCATGTGTGTGTGTGTGTGTGTGTGTGTGTGAGCGTGCGTGCATGTGTGTTTACTAGGGAGAGAATGAGAAAAGGAGGCTGAATCATGCTCGTCCTGCCAGTTAGCCCTGTCTTAAGCTTTCCATCTAGCTGGGACCCTCGGACGCAGCCCAGACCTGATAGCCCGAACCTCTGCCTTTCCTCCATTTTCTTTGCTTTGGTTTGTTATAGTGCACATCTAGGGCATAGCTTCAGCTATCCATTGCAATGCCTAGGTGGTGAGCCATGTGCCTAGCTATAGGTCTTGGGAGCAAACAACTTGGAATGAGTGCCTGTGCCACTGACGGAAGGGCATTGTGCAAATATGTCCCACAGCAGGGACACTGTCTGCCTGCCAACTAGCAGGCCATTGGTCTTTCTTCAGGCCATAGGCTGGGACTGGACCAGCAAGGCAAATGCTACAGGGATGGGAAGCAGAAGGAAGCCCCCATAATCCAAGGGGAAATGATTAGCGACCTGCTACACCGCTTAGACAGATTCAAGTCTATGGGGTTGGATGGATC |
| Pha39 | Osp107_A07 | GATCTGTGCTGGCTGCTTTCCAGCAGGGGATATACATAATGGCAAATGATGTCATGTAGTGGAGAATGCAGAAAAATTCTTTAAACAGCAAAAAAATAGGGGAGTTGCTGAAAAACAGAAGTGTTGTGAAAATGTCCTATGTATATGTGTATGTGCGTGTGTGTGTGTACGCACGTTTATAATATATTTCTGGCTTCCCGTTTTCGAGGGAAGTGGCAAGAGACAATAATATGTTTACACAATCGGCAACTGTGGCAGCAGTATAGAACGGGCAGAAAGGGATGAAATGCATTTAGCCTCTGCCTCTTCAAGCCTTTCATTAAGAGTATTGTAGATTACTGCAGCAAAAATGTGAGTCAGTTAAAAAACTGACATTAAACAATGCCAGATGTGCTTAAGTAGCAAAATATCCAGAAGAAATTACTAACATACAAGCTATGTGTGGATTAATGTAACTGCCTGATC |
| Pha40 | Osp107_B03 | GATCAGCATCATGTTTAAAGCAGTACAGAAACAGACCTGGATACACATCTACACAACATCCCCACGTACACTTTTAAAAAGGAATTAAGGATACAAATAATGCATATTTCTGGTCATCAACTAACTATTAATTGCCTGAGACAAGAAAGAAAACTCCCTCATCCAGACGTACTGAGTAATTGCCATCTATGGACTTTTTCATTTTTTTTTCCCCAAAACACCTAATACAAATTACTATTAGGCACAGCATACGGGTCAGAATAGAGAGGAACAATGTTTTAGAAGATAAATTCTTGTGACCAAAACCAAATTTGTGCCTTGCAGAATTATAGTCCTTAACTTCAAATATTTTCAATCAATGATTACCAAATAATAAAAAAAAAATAGCAGAGGTCTACATTACAACCTATTTGAAGAGAAAATAACAAAGGTAGCTAAAACCCAGACAGAGTCTAAGGTCTCACTCTTTCAACTACTACTAACTCTGGAGTTGAGCACTATCAGATC |
| Pha41 | Osp107_B11 | GATCTGCTCTTAGTCCCAGACTTGGTCAACGGTTTATGTCTAAAGGGATGAGATGTAGGGATTATGTAAAAAGGCAAAGAGAAAGAGAGAGAGAGAAGAGAAAGATTTCACTGGTTCCAGCATTGGTTCAGTCAGCTAAGGGGTCCGGTTCCGGTGGGCTTGTGCACATGGGGCTTTGGTTTGTTAGTCATCAAGTAAAACTTGCCCCACCACAATGTTTGAGACATTAACTCTTTCAGTCTCTCACAAAGGCGTTTCAAGTGCACGGATTTCCCATTCAAGCAGTCAGTAGGTGCTGGTTTGGTTCAGCCTGGATGTTGGCCCCTAGACAGCAGAGTTCAAAGCTTTCTAGATTACTTGGCCAAACTAAGGTTTGGTCCTTTTTAGCTCGCTCCGTTGTGTTCATTTTGGCCATTATTTTTTCTTCTCTCCTTCCAGTTTTTTTTTATTTTGAAGAAGCAGCATGGTGGGTTTGGGTGTTTTTTTGTGTGTGTGAAAGTTCTCCCTTTTATTTTGCTTTCCTAGTTCTGTGTCGGCCCCGTGCCTCGAGGTGCTAGAAGGTCAGTGAGGCCATTGACTCTGAATTTGGTTTTTTTTCCCTTTGGGGATC |
| Pha42 | Osp107_C01 | GATCAATTTAATATTTTCAGCATTGCTTTGCCTGATTAAAAATTGACCTTTAAAGCAGTTTCATGTACTGAAATAACATTTTTTACCAAAAAAAACCCAAACAAAACAGACAAGCCTGATGGTCTTTTAGGTTTGTTTGTTTTGCGTTTTTTTTCTTTTTTTTTGTGTGTGTGTGTTTGTTTTGGCGTGTGTGTGTGTGTGTGTGTGTGTGTGTGTGTGTGTTGCTTTTCTGCTTCCGTTCACACTACTGCCCTGTTCCCACTCCTGCCTACTCCTTAATTCCAGTTACATACCATCTTCTCTAAATGTCTTTGAGGTCAATCCTTTCTCAAGCCTTTTTAAAATGAATGCTTCCTTTCAGATC |
| Pha43 | Osp107_C07 | GATCCCTCCCAGTCCTTGTCTCGACCCACGAGCCTGCCTTTGTATTTTCTCCTCATCCCAACGTGGCCAGGCGCGGGGGTGGGGAGTGAGTGAGCGGCTTCGTGGTGCTTTGTTACCGACTGAGCTGACCTTAAACCACGACACCCACTATTCTTGCTTTTTATGGTTAGGGACTTTTGTTTGTTTGTTTACCTACATGGTATCTAATAGAGGTCATCACTGAACTCACACACACACACACACACAAAAAAAAAAAAAAAAAAAAACCAAAAACACCACCAAAAAACAAAACACAAAAAACAACTTACAGACTAAACATGGCCTTATTTAATATTCCCTGTGCTTGCTGCTCCCAGCCTTAATGTTGCAAATGTGGCTGCAATTTCAGCTTCCATTCATCTAAAGAATCAGGTCATTCCAACCACCTGAAGAAATACATATACCATTACCAGGCATTTTAGCATAGTCAAACATCACTGGCATTGAC |
| Pha44 | Osp107_C08 | GATCCGACTATGGGCGAGGGCAGAAGGAAGCATGAATACACACACACACACACACACACGAAGATATACCCGAGCAAACTTGCACACGAGAGGAAAGCAAAGGCAACGTGGTTTTTCCACCATGCCGTGCCATTTCTCCGCATCCCCTGTTCATATAGCAGTGATTGTGCTGGGTGTTTTTCCTCTCTGAAATCCTTTTCTATGTCAAGGCTACACTGAGTCTATAATTAACTCTTCATGATGGAAATGCGCCCTCTCGGGTCACTTCTTCAAAGAAATTAATTTTTAAGAAAAGTAAATGGCAGAGAGAAATATCAGAAGGGGCAGTCAGAAGAGAGTGATC |
| Pha45 | Osp107_D01 | GATCTGAATCATTCACTCGGGGTAATTAATACTGACCTAATTACATGGATGAGTTTTGCACTGAAATTTTTAATGGTACTTAATAATTAGAAGTGTTCTTCATTCCCCACCTCTATGATTCAGTCCTTTGCCTTTCGTGCCTCCAACGAATAAGGGCACTTGATGAATTGAATTCCCTGTTTATTTCGTGTTGGGTAACTCTCCAAGTCCCCCTGCCTGGAGAAGCAAAGGAAGTTGCTGCAGTTGAATATCTCTGCAGAGAAGTTCTAAAAAGGAAGTTATAAAAGTAAGCAAATAGCAGAGGCTAAGAAATATCCTGCAGGCCCGGTTCCCCTCACTCTGGGTTTTTACAAGGACAAGATGGTTTTAATTCAAAAGAGAGAGAGAAAGACAGAAAGAAAGAAGGAGAAAGAGACAAAAAGAAAAAGGCAAAGATAAAAAGAGAAAGAGGGGAAAAGAGAGAAAGAATGCCAGAATGATTGAAGTTGGCAGGGACCTCTTGGGGTCATCTTGTCCAAGCCCCTGCTCAAGCAGGATC |
| Pha46 | Osp107_D03 | GATCTCCCGCGTGCTAGAATCACGTCCTATCTGCTCCCAAATGGGCATCACCAAGTTGGCTGCTATGCACAGCATAGCTGCTGCCCTGACTATTCAGCTTTCAAAAGTAGAGACTGTTCAACAGTAAGCCTCAAACTATTGCAAAAAGTGACAGGAACAACAACAACAACAAAAACCCCCAACAAACAAAAACCACAACCCTCTCCTTCCACCCAGAAAGTGCTACTCCTGTTTCTACCACAGCCCTGTTGCAAAAGTGGGCACCAGTCACTTTGCTCCAATATCCTTGCAACTCTAAACTTGAGGGGAAGGAGATGACAACTGGGACTGAAGCAAATAAATTCAGAGCAGAGGCTGATGAGGAAGGACAGCAGAACCTGAGTACTCAGTATTGCGCATAATAGAAAGCTTGGGCAAGGAATTTACTTCAGTTCTCGCTTGCAAAACCTGCATCAATGCTATGATGCCATTTTAAATTTAACCTCTTGTGGGCTTTGTCTTCAGATTTAAAAGCTTCTCAGAGCACAAGAAACATAACGCAAGAACCAACTCTTTATAGAGCAGCAACACCAACCCATGCAGGGGTTAGCGTATCTCAAGAAAATACCAGTGTTAAAAGATC |
| Pha47 | Osp107_D04 | GCGGTACCCGGGAAGGTTGGCATCCCTTTGTCCTATGAACTGTGGCTTCTTCTCCCCTAGGAATTTTGAATTACTGGAGGTGAACGTACCAGCTCTAGGCATTAGTTACTTTTAATATAGTCTTTTATTAAGTATTTTCTTTGGAAACATGAATCTTTTAATGATTTTTAACTTGTTTAACTTTTTGTCCTTGTCTTGTCCTATTGATATGCTCTTGGGGTTTACTGTCCATTTCTCCTTCTGTGCATTATGGGAGAGAGACTGCTTTTCTTCTCAATTTATCCACATTAAGGCATCTGAATGCATGAATAGGTACCAATATTATTTTTTCCCTGTTGTGTGTGTGGTGTACACGTGTGCGTGTGCATGTGCTGTGTTTTAGGCTTTTTAGTATGCTGTAGTCATCCTAATAGCTAGTGCTTAAAAAAAAAAAAAAAAAGACTCACTAGCTTCTGTGTCTGCTAGCAGTGACAGCTACAGGACATTGGCATGAAGTGGCTTTCTTGCATATAAAACACGCTACTAATAAAGCACTGAAATGTATTTGAATTTTCCTTTAACCAGTTTACATATTAGGAACTTAAGGATGTCGTGTTTGTTGATGATAACACACCATCTCCATTATCCCTTCTGAAGCTATGAATGTTTATGGCTGTGATAGCTTCAGTCTGTCAAGGGAGAATGGTGCAACATACTTTGTATTTTTACACCATTTTGACCTACAGCCTTTTAGGCAATTCTTTCAGCTGTGTTCATCATGCACCGCTGAAAGAGAACCTGAAAGGCAAATGTGTTATGTGATC |
| Pha48 | Osp107_D05 | GATCCTAGTCTCACCTTGCAGCATGGATACTGGGAAGCCAAAGCAGTGATGGTGGCACCTCTAGGTAAAACCAGACCTGAGCCCTACACATGCCAAACACTGCGCTCGCCTGCGGAGCCCAAGCTCTCCGCCAGTCAAGTAGCTGTGCATTACCAAGCTAAAATTTACTCTGTGTGTGAGTGCAGAGCTCTATCATATGTGTTGTGGGTGGTGTGCTCTCCCAGCGTGTGACCCACCTGCCAATATGTTAGGGCACTCTGCTCTGTAGGTTTTTTTTTTCCTGCTGCAGCCTGATTCCTACATAAAAAGTCATTTGCTGTTTTTTACCTCAAAGCCCTTTTGATGACTTTGTGACGAGCAGTTTTTGAATTGGGGTTTTTTTGTTTTTTTTTTTTTTTTTTTTTTTTTATGAAGGTGTTATTTCTTGGAATTTTTGTTTTCTTGCCAGATAATGGAAATGAAATGATGCTTATGCTAAGAGAAAGGATGTTGTTGAGAAGGACAATTTTAAAACACTAGGTGATATGCAGTATTTCTGAAAGACATAAAATAAATCCTTTTAACTGAAAGATTTTCTAAGCTGTTATAATTTAGAATTTGTCAGAGAGGGAGGGATGCACTTGGAATTCAGGTTCAGAAACTTAGGTTATGTAAGTTTGCCATGCTACTGTTTTAATGAAAATAATGTATACATCAGCTATTGTATGGTATATATCGATGACTGATC |
| Pha49 | Osp107_D09 | GATCTTGGTTTATTATTGTAGTGAAAAACAAAGCTCCTGTTTGTTTTTTGTGTTTGGTTTGGTTGGGGTTTGGGTTTTGTTGTCTGTTGGCTTTTTTTTTTTTTAATAGGATTGGCTTTCTTTGAATATCTATTATATATCTATGAATTTAATGTCATGCAAGCATTAATAGAAGTGGGGTGTGCATTAAGCATAATACTGGCACTCTAGATTATATTGATTTACTGCCATATCAGTACAGCTTTAGGAAGGGACAGCTCAAATTTGGCACCTGTAGCAGTCAAAATTTGATC |
| Pha50 | Osp107_D12 | GATCATCCACACAGAAAAAAGTGTCATTTTCCAAGATGATATCGCACAGGAAAGCAGTAATATTGGTACCGTACCCGAGAAAACTAGCATCAACTATTTCCATTCTTCAGTATCCACAGGTTAGCTTTGACGAGGAGGAGGGTGGGTTTGTAACTATCTGCCCATTCTTTCAGTGAACAAATAAATTTCTGAATGAAAACTTCAGGAGAAATACCAGAGAAGCAGTTGTGCAAGGAAGCTCTTCTAACTCTCCGTCAGGAGCACCCAGCACACAGAGTCCTGTTCAGGCTGAACAAGATTTATTGCAGGGGCACACAAGGCAGTTCAGAAAAGCAAAGCATGCATACACATACACACCTACACATATGCATGCACATCCACACACACACACATCCCCTCTGGTACTTTTTTAGATC |
| Pha51 | Osp107_E03 | GATCAACCCCATCATTTACGCCTACAGAAACCAGGAAATCCAGAGGTCCATGTGGGTGCTCTTCTGCGGCTGCTTTCAGGCTAAAGTGCCCTTTCGCTCCCGGTCCCCCAGCACTGTCTGAGTGACTTTTCTCTCTGTCGTACCACACCGAGTGACAATCTACCCAGTGCCTGTTCTAAACTTCAAACTACGTCGTGAAGTCATTGGAAACATGCTTAAGTATTAGCACTTTATACATATTTGTATCTCTGAGAGTGGTTCGGGGTATGGAGTTTTGGGTTCTGTGAAAAAAAAGCGTGGTTGTATATAAATTTGCACATCACATTTGTAAAGTGAAGACATTCCGATACTGCTTAATTATAGCACCTTATTTCTAGCTGCTGACCTGCCAAAACAGTGTTGCCTTTCCGAAGGGCAGAGAGAAAGAAAGTTGTGTTTCTGTTGTATTTGTATGTGTGTGTGTTGGGGGTGTGTGTGTGTGTGTGTGTGTGTGTGTGTGTGTGTGTGTGTGTGCGCGCGCGCGCACGCGCGCGTAATGTATCTGCTCTGCATTTCTTCTGGACTGGAAACTGTCTTAAACTATCCAATTCAATGTTTCTCCTATATAGCCTTCAGTTGAAATTACTGTCTCCTTTCTATGGAGCCAAAACCATTGTTATCTAAATTCCCTGAGCGAAGCAGCAGGTATCATGGTGCTAACCACACATTTGGTTCAGG |
| Pha52 | Osp107_E05 | GATCTTATAGCATAGCTGACTAAAGCTGAACATGAGTTAGCCAGTCCACTTCAACAATGATGTCACCCAGTTCTTGCTTTCTACAAAGTTATGATTAATAAAAGGGGAGGATTATTACAGTAACTTACTCTTAGTGGGCAAGGGGTGACATCAACAGCAGCTGAGCAAACTGACCAAACTTACTTGTGTGTTGTTGAGGTTTTTTTTCTGTAGCAATGACTAACAAGGGGCTAACAGCATCTGATGGCACAAAGGGCCCTTGAAACTGGAAGGATGCTTCTGGTGGAACTTTTGAATAGGAACCACCATTTTAACTCTTCTGCTTCGTTTTACAGTTGCAAGATGTTTCAGCTGGCCTACAACAAGCTCTTGCCTTTTTCAATTCATTAAATACACACACACACACACAAATGCAAAACTGGTCAAAAACTACCCCAATGGCCTTTCCAGCGTAAAGCTGCATGATC |
| Pha53 | Osp107_E08 | GCGGTACCCGGGAAGCTTGGAGTTTTCAGGGCTGGCCCCTTCCTGCTGGCAGAACTAGCTGGCTGGTTGCTGGTTCAGAAACAGTTTCTGCGAGAAGCAAAAAAACATGCTGTCCCCTCTGTGGGCAAGCAGAGTTTCCCTGTGCGTGCGTGCGTGTGCGTGTGTGTGTGTGTGCGCGCTTGTGTGTGTGTGCGAGCGTGTGAGTGTGTGCGCAGGATC |
| Pha54 | Osp107_E12 | GATCAGCCATAGAGGGGAAACAGGATGAGGCTGCTGGTACTGCCTGAGTTCATGCGAGTGCCGAGTATGTCTGCCTCTGTTAATATGTGTGGCCACCCATATGTATACATATGTATGCAGTGTATATATGTGCTCTGTGTGTGTGCACCTACTGGGAAAAGCTGCTCAGCCTTGCCACTGCCTGCATCAGGGGCACAGAGATGGGGCAGCCTTACCTCTGTCAGCTGCCACATTCAACCCTTTCTAACACAATCAATTGCCTCTGAATTGTTAAATATTCTGTAACTTGGCAGCTAAAGCTCAAAATAGAGAAAAAAATGGCAGCTAAACTGCAAAATTTAGATTGATTTCCTTTAAACACAGATC |
| Pha55 | Osp107_F01 | GATCCACCACTCGTAACAGGCACTGCAGCGACTTGTAAATGCACTGCAAAAATAACTTCTTTGACCCATAGATGAACAACCAGTTAAGTGAACCAGTTGCAAAAAGAAAAGTTTTTAAATAGCTGCTTGAAAACAGGTCTTTAAAATGGTTTCATCACGGTATAAGGCACCAAGTAAAACCAGTTAAGTGTTCTTTATAAATTCATTGACAAACGTTTTCCTTTCTTTCTGCCACCTTGAAGAGAAATAAGTCTAGTATATACAAATTAAACATTACTGTGCCTGAATGAATCACCTGAATTAGGTAAAGACTTTACCTATAAAGTGTCAATAACTTAAGATTTTGAGACAAGTAACTCCTCCTATGTTTTCTGCAATAGGCAGCACTTTTAGTACCTCTGTTATTTTGCAAGCACTATGAAATAAAGCCATCTGTTGAAAAACTGCTGACTTTGCCATGACAAGACTGAGGATTAAAACTAAAGGAACAATTTATAGGTCTATTCCTAAACACACAAATCCATTAAATATGACTTATTTTCTCTCTCTCTCTCTCTCTCTCTCTCTCTCTCCCTCTCTTCCTCTCTCTCTCTCTCTTCCTCTCATGAACTTCTCAAAGCAAGATTATCATAGAATCATTTAGGTTGGAAAAGACCTTTAAGATC |
| Pha56 | Osp107_F04 | GATCATGCCCACAAATAACCTGGGCTGCTGTGCTGTTCCCCGTACACCTGCATCACCCACAATGCCAATGGCAAAGCCAGCAGCCAGACCACTGAGGCCCACACTCAAGCCAGCACCCAGCTGAAGAAAGCTCCTGTAGGACAGATATGAAAAGAGTTTCAGTGGCACATAACAGGACACCCAACCCTTCAACTTCATGCTAGCCTTTTTTTTTTCCTCCCCAAGGCAAGTAAGAATTAACATAAACATTTAAAGGGAACCTCTTTCCTGCAGAAAGAGCTTCTATACTCTTTAAACAAGCACAGTATGTCTCACAAAGCAGATGCTTCCTGAGACTCCTAGCTCTGATACTTAAAAACAGGCAGCTATGCAAGATC |
| Pha57 | Osp107_F07 | GATCAAGCCCTTAGCAAGCATACAACCCAGAGCTTTTATTCACATGCAACCACACACAGTTAAACTGCTATTAAATTGCATCATGCTGCTTATTCTCCTCTCTGTATTGAACCATGAAGTATCAGATTGAACAGTTCTAACAAGCAAGTCTCAGCCCTACGATAGTAGCTATCATAATAATCCATTCTACTGGCATATTTTCCTGTGGTTACTTCAGTTATGACTCAACAGCTTTATATAACCACACACATATATTATATTTATATATATATATACACACACACACACACACACATATATATATATCAAATCAGGGCCAGTGTCACAAGACTGGACACACCAAGCTTCCCGGGTACCGC |
| Pha58 | Osp107_F11 | GATCCTGGACTTGTGTGTATTTAACACCTCCTGTTGCGCGCTGTGGGGAGAACTCCTCGAGGATGCTAGCTTTTATAGTTCATTCGAAGTCCTCCAGGAGGTACATACAGATTTAATCTTCTTCTCCACAATTTCTCTCTTGACTGAACTAGACAGTTTCTTTTCCTGGGAGGTGCCAAGTTTTGTCCTTAGACCAAGTGCCAGTACTTGCCCAGTTAATTAACAGCTCTTTGCTTTGAAGAGATAAAGGTCTCCCGCATGCACACACACAGACAAACACACACACAGAGACACACACAGACACACACACAGACACACGCACGCCACAGACCTGCTCAGCGGCCACTTAAGCTGATC |
| Pha59 | Osp107_G09 | GGGTACCCGGGAAGCTTGGGGTGAAGGGAGCAGGCTGGGTGTAAATAGGCATGAATTGGGGCTGCTGTGAGGAGAGGAGGGGATTCGAGGCTGTCTGCAGCTGCGTGGTGTGGGCGATACTACACTGCCGAGTCCTGAAGCCAGCGCTTGCCTTCGGCTCTTGCAAAAAATCACTTTTTTTTTAAGTAAATAATAGCATAAGTGCGTGTGTGTGTGTGTGTGTGTGTGTGTGTGTGCAGGTACAGGTCGCGATC |
| Pha60 | Osp107_G11 | GATCTCTCTCTCTCTCTCCCTCTCTCTCTCTCTCTCTCTCTCGATATAGAACAATAATCTTTGAGAGCAAGATGCATTTTATTGCTAAAAAAGCAGTTTCAGGTCACTCCACATCTAGCAATAACAGCTGTATTTAGCACAGTACTTTGTCAGAAGAAAATAAAAAAAAATCAAATGCAGGGAGGGAAAATCAAAGAGTTAGTTACAGTCTGGATGTTTTAAAGCCTAGTTTAGCTAAATTCAGCGATAATTTTTCAAAGGAGAAAAATACTGCAATAAAACATTTATTGTGTTTTTGTGCTTTTTTTCATTCCTTTGAGAAAAATGAACCAGTTTAAGACTCATCCAATACTAATTTTTTTCTTAGTTTTTCAGTTCAAGCATTAAAACAAAAAATCAGTTATTCACATAGTTCTCGCTATAGATTATACTAGAGGAAAAAGTTATAAGATAGCTTTTTGTTTGGCCTTACCCAGTATGGTTCAAGAAGACATCATATGGTTTAAGTGGACATTGGCAAGATC |
| Pha61 | Osp107_H02 | GATCCAAATGCAATTGAAGACACACACACAGAGATACCAGTACAGACGGAGGTACAGGTACAATTAAGCAGGGAGGTACCCATACAGATACCATCACAGACACTGATACCAGCGCAGAGACCGCGATGCCGTTACAGTCAAACCCATACCGAGACCATGAGTGGTACAGAGACCATTAGAGATAGCCTTGCTGATACTATTTCAGCTAGCGAGTCTTTGGAACTTAAAGGTGGGCTTTGGAACCTATACATGAGGCTTTGGACACTAAAAATGCATCTTTGGCTCCTAAACCCGAGACTTTGGAAACTAAAACTGAGCCTTTGGACCATTAAAAGGAGGCTGCTCTCTAGAAAATAGGCTTTCTGCACCTTAAGATGAGGCTTTAGATC |
| Pha62 | Osp107_H03 | CTGTGTGTGTGTGTGTGTGTGTGTCTCTGTGACACATGCTCAGCTTCTCTGCTGGTTGAACCTGCAGGTGGGAAGGCAGCAAAAGCATCCCTCAGCCTGGACAAGAAATACCAGGGCACCAGGCTGGGCTCCAGCAATAGAGAAGGACCATACTGCGTGGTACTAGTTTGTACTAGGTGGTACTGAGCTCCAGCAGCCAAACAGGAGGGAGTCCTGGGACCAAGTGGCTGGAGGAGGAAGTTGCATCTTAAGTCATAAGGTGCAGTCAGCAGGGGTTTATGAAAGGCAGGTCCTTCTTGACCAACCTGGTCTCCTTCTATGACCACGTTACCCACCTAAGGGATGAGGGGAGGGCTGTGGATGTTGTGTACCTGGACTTCAGTAAGGCCTTTGACACTGTCTCTCACAGCATACTCCTTGAGAAGCTGGCAGCACGATC |
| Pha63 | Osp107_H06 | GATCCTCTCCCCCAAGCACACTGAAGTGCAACAGTAAATCAGGTAATTACGGGAAAAATATGGGCAGCATTTCCAGCTGCCAGTCACTCCTGGTGGCTCGTCACACACACACACACACACACACACAAAGAAAAAAAAAGAGTTAATGAATCTGGAAATTTAGTAGAGAACAGGAAGCTGGGAAAGCTAAAATGTGGGAGTGCATTGCATCCTGCTCACTTCTCCTCATCTGTGGATGCAGTTGCCGGTTCAGAGCCCCGGCGTGCAGCAGCTCAGGTACCTCCCGCTGCTCAGAAACCGACGAAAGCCTCGTGTTAGACTCAGCCAACGGTTTCGGCACCGGGAGAGCGAATCACTGTTCGTTCGTTCCCCATCTAGACCTCAGATC |
| Pha64 | Osp107_H07 | GATCACCTCCCTCGACCTGCTGGCAGCACTCCTCCTGTCGCAGTGCAGGATTCCATTTGCCGCCTTTGCCACAAGGGTACATGGCTGCCTCACCTTCAACGTGGGGTTCATCAGGACCCTAAAGTCCTTTTCAGCAAAATTGCTTTCTAGCAGTGTTGTCCTCAGCATGTACCTTTGCACAGGGCTCTTCCTCTCCAGGTGCATGTCTCTGCACTTGTAGTTGTTGACCTTCATGATGGTGAGTGTGTGTGTGTGTGGTCTGTGTGTGTGCATGTGTACACATGCCATTTAAAAAAGAAGTTGTTCCTGTCCAGTTTCCAAGTTGGATATTTCTAGGACTTTATGGGAGTGTCTGTTTTTCAGATC |
| Pha65 | Osp107_H09 | GATCATGTTATCCTGAGTTTGGGGCTTGTTTCAAGCACTTTTATTTCTTTCTTTACACAAGAGACAAAAAAACCCAGACTCCAGATGAAACCAGTATGTGAAGATGGGAATGCCTCTTTTTGTGTTTGTGCTTGTGTGTGTGGTGTGTATGCACCTCTAATTTTTCATGCCTTCTGGCTGCAGGCCTTTCAGATACAAAGCCCAGGCTGGTACAAGGAAGCAATCTTTGTTGGGGAGCCACCAGCCAGGAGAAGAGGTAGTGTTCGGGTTTCTTCCCTAGGGAGACCTATGGTGGGCCCTGAGTCTCAAAACAGAGAGAGGAAAGACCCAGAGCCAATTCCGTGGTCCCACAGCCTCTCTGCACTCAGTTACCCCTTATCTGCCTGTCCAACACCTTTAACTTTCTAAAACCTATGACTCAGTTACAGAGCAGGGACTTCCCAGGATTACAGCCCTCACAGCTTTAGGAAGAGGTAGAGAGGGCTCTCTTCTTAAAAGAGGACTTCTTATCTTCCCTTGGCCTCTAGTTTCATAGTGAGGTCCTATATATCAGTGGCCCTTTTTCCACAACCACTACACATCTGTTTGTGGGAGAGAAACCACTGGGGCTTTTCTGCTCTGCAACTGACTGGACCATGCTTGGCTGCTCCAAGAGCATCTACATGGTTCCTCTCACAAAAATACCCACACCTGGTTTGATGGCAGAAGGGGCTGGCGACACATTCTTTTGTCCTCTCAGATC |
| Pha66 | Osp107_H10 | GATCCGTGAGTGCTCAGAAAAAAAAGACATACACACACACACCCCCTCCACACACCCCCATACACACACATCAAGAAAAAAAAAAAAAAGAACATTTGGGGGTGAGAAACCCCAAGGGAAGGAACTCAGATGTTCGGGTCAGGCCTTAAAAAAGGTATCAATTCTTCTTTCCACTTAGCACAGCCTGCTGCCTGCCCAGGCTCTTTGTCTCTGCCCTCTTCCCAGTACAGAAAGCATTTCCCTTTTAAATGACATCTGAGCGCTGACATGCTGGAGGCACCCATCCTGCATCATCTCTGGCAGATGGTGCAGCTGGCCACGATTTTCGGGATTGCTTAAATAAAACCACAGTGCCATATCAAGAAATGTAGAGAAAATGCTCAACAGTGCTTAAGATGTTTAAACATCACAAACTGTCTAAGACTTCAGAGTGCTGTACTGCGCATTTTTGGTGATGTGACTATTCTTATCGATGGTAATCGTAAAATCTCCATCACGATTCATTTTCCTTGCTTTCTGGGAGGCAGAGACAAGTCCTACAATACTCCTGACCCAATATGAGACTCTTTCGCCTTGCCACATTAGGAAGAAGTTTGGACAGTTGTAACGATACAAGTCAAACGCTTAGGGTTTCTGGAGAAGGGAGCTTCACTGCTGCAAGCGTCAGATC |
| Pha67 | Osp107_H12 | GATCAAATGCCCTTTGCAGCCGTACACTATCTGTAGTTTCCTTCCTGCCGGGAGACAGCCTTGCAGAGAGTTGCAATCAAGAGGAACCTCGTCCTCCACCAAAGTGTTTCTGATTGTTAGGGAAGGATTTAGTCTGCGCAGCGACTGCAGACACCGCAAGAGGCCTTAATCAGGGTCACGATGCCTGAAAAAATGCCATGGGTGGGAGGTTCTGGATGTGTAATTATGTATTTCTGTTGCATGTGAGCGATTTAGATTGCTTAGAATTAACAAACATGTATTAACTCCGAACACATTTTTGGTTACAGTTGTCAGGCTGTAAAAGAAGCAATGCTTCTTCAGTCCCTGTAAACCTGCTTTTTGTGTGTGTGTGTGTGTGTGTTGGTTTTTTTAATGCTTGCCTTGAAGATC |
| Pha68 | Osp108_A01 | CGGTACCCGGGAAGCTTGGGGTTTTGTGTGTGTGTGTGTGTGTGTGTATGCAGTTCTTGTTTTGAAAATGCATCATTTTTCAAGTCAGGTAGAGATACATTGTTTCTGAGAAGGCTTCTTGTACTCATATGATAAAGCTGACCAGCTTTTGAATTCCATAAGAAATTGTTGAACTGGATC |
| Pha69 | Osp108_A03 | GATCAGCACCTCCCCTCCACTGCCTCCCTTGAGGAAGTTGTAGACTGCAATAAGGTCACCCTTCAGCCTTCTCTTCTTCAAGCTGAACAAGCCAGGTGACCTCAGCCACTCCTCATAGGACTTGTCCTTGAGACCTTTCACCATCTTGGTCGCTTTCCTTCGGACACACTCGAGTAAGTTCAATGTCCTTCTTATATTGAGGTGCCTAAAACTGCACACAGTACTCGAGGTGGGGCTGCATCAGAAATTTTATGAGAGTATACACACACACACACACACACACACACACACACACATTTATGCATATATCTATACATATGTATACATCCAGAGAATGATC |
| Pha70 | Osp108_A04 | GATCCCAAATGTAGTTATATAGAGGACTGGAAATCGGAAAGGACAGGGGTTTTGGCATTCTGTTTGGCAAAACCCTGGTGTGGGACCCACTTCCACTAGGTCAGTAGAGATGGAGCATGGCTGATAGGAGAGACTGAACTTGCTTCACACCCACTTCACTGCCATGTTACAATAGCTGGTTTGGGTTTGTCCGACCTCCAGCGGGCTCGGTGGCACCAGCAAGCACGCTGGATTGCAGCTGCCCCAGCCCTTTGGGCTGTGAGTGTGGCTCACAGAGGACAGGCTGAGCTCCAGTCTCAGATAAAACTCCCCAGCAGCAGGTTACAGCACCACGTACACAATTTATATACACCACATATACCATAATACACCCTTTCACAATAACTCTTGAAGTAGTATTATTCCATTAGAACCTCAGCTTTTGTTTAAAAATTATGTTCCTAACCCTCATGAGCTGTTTTAGAAAACTGTCAAAATCTGAACCATGAAGCCTTAAAAAAAAAACACAAAACCCTACAAAACACAGAAGGCAAGTAAAACAGTCTGAAATACATCACTTCTAAGATC |
| Pha71 | Osp108_A10 | GATCTTCTCCTATATATATTCATATATATATACACACACACACACACACACACATATATACTTGAAGCACTTAAACATAACACTACTCTGAAGTTTGGGACAACTCAGTAAAGAGAATTCATAAACTGAAGCACAACAGGTATTTTTCAGTGCTAAAACTGTGTCCCTGATACCCGACACTGACCAAAGATC |
| Pha72 | Osp108_A12 | GATCTGGGCATCCTGCGGTCTCATCCTACAGGACTGGTGAAGGAGAAAATCAAGTCATCTCTGCAACTCCCCTCTCATTCACCAAGCCTCCCAAACCATGTTCCAATGAGCTAAGCTCGAGTTTCCGTGTTTAGAGAGCAAGCTGAGAGACATCGTTTTAATTAAGAAGGAGGTTCCAGGATGGACAGGCATTGCACAGAGGTAAATGGTTGTGAAAAGAAATCACTGCATGTGTCACAGCATGTAAAATGGACATACACAGAAATTTTACATTTCAGCTGCAAACCAGCAAGGTGGCAACCCTCCCCATCTCCAAACCCACCACACCTCTAAACATATAAACGTGTTATTAGTTTCTTCATACCCTGTAAGAGAGAGACAGCCTGTATCTTTGCTCTGTAAGCTTCCACATCAGCTGCAGAAAATTAGCGCCTTAACCTAAAAAGGTAAGGGAAGGAGGGAACATGAAAGACAGACTGAGTCAGCAAGAGTGTGTGTGTACATGGGTGTTAGTGTGTATGTGTGTGTCAGACAGACAATGTATCTTATTCTGGGTGAGAGTGTACGTGTATGAAAAAAGACTTTCTAGTGTAATTTATTAAATTACACTAATTAAATCTGTGATTAAATCTGGAGCTATACTTAGTACCGTGATAGAGATGAGCAATTCAAGAGTTTAAGCACAGCTCTCCAGCTGAGCTGTGTGGTGCTTGGGATC |
| Pha73 | Osp108_B01 | GATCACCACTCGGATGGTGGCCAGATTAAAGGGGAACAATGCAGTTTTAAATGTAAAGTTTGCATCTAATTTTTTTCTTTGCATGAGAAAAAGCACATTTATTCTATCTATGTGTCTGTCTAATGCTCAGCCTGTAGTTTGTCAGTCTTTTCTTATTTTCTTTGGCAACGTGAGGCATGCTCACGCCTGTGTGTCTAAACTGTCTCTCCTGCACTTGTCTGGTTAGGGCTCTAAACAAATACTTTGTACTGTGGGTGTGTGCATGTGGGTGTGTGCGTGCATGTGTGTGTGCGGACAACACTGAATGCACAGATTTTAAACATTATTCTGCTGCTAAGATACTGTATTTCAAGAAGCTCCTCTCCAAGTGAACAAACAAGCACGGGAGAAAATGTTCAGGAAACAATGTTTTGATAGAAAAAATGGAACTATGCAAAAATGCTGGTAGCGGAATACAGGGAAACGCATTGAGTTGGGCACCTGAATTAGATACCATGAAATGCCACCATGGAAGTAGTTTCTTGGGATTGCATCTAAAAAATACTAACCAAATGATAAACAGTCATCTCCTACTATAAAACTCAAGGAAGGGTAGAATGGGAACGTAAGTTAGGAGATC |
| Pha74 | Osp108_B03 | ATGATTTAACAAACAATTTTGACCACAGCTTTCAAATCAATAATTAGAATGAAAAATCTCTCCCCTCTATAAATATGACATTAACATAATTATACCACCCCCATCGCATAGACCCATCCTCTTTAGGGAATACATCTGATGCTCTGAAGATGGAAATCAGTCAGATTTTAGTTGCTCTCAAACGTACCTTTGCTTCTCACTGACTGACTGGCTTCACCTTCCATGCACACACACAGTCACTAGCACACACACGCGCACACACACACACATACACATGCGCACGCACATACACACACACACGCACAAAAAGGCAAGGCAAAGCAGCGGATGCTGACTCAGAGCAGTTGTGTGTGGAGGGGGTGGTATCGGCTGGTATTTTCACTGCACTCATCATTAACATTAACTCACTCTCTAGCATCCACCCTCTCTCAGACCTGCTGGACTGCAGCATTTGCATGACGCTGTCACTCTAGCCCTTGTGGGTCAGATGTTAGAGGAAAAAAAAGCCTCAAGGCTCTCATAAGGAATGCCTTCATTTCTTCCAAAGGGTCCCTCTTCTTATTTTTCCACCTTTCTACTTCCTCTGTTGCTGTGTCTCAGTTGTTTGACATCTTTCCGGAAGAAAATGTCTTGAGCTGCAATAAGCCAGTAGTCAGTCGGTCCTCCCCTCACCTCACACTCCTATTCCCACCCCCTTTTTTTCCTAATCAATAAGACAGATC |
| Pha75 | Osp108_B04 | GATCCGAAGATAAACAGTTCAGTGCCCTCAGTGGATTGGAAGGGTCATAAACATGAACAGTGAAGCCTGAAGGTTTGAATTGCTCTTGCTGTAGACAAGAGGAAGTTGTGTGTTCTGTGAAGAATGCTAAACCAAGGATAAGATGGTGACTATATATAAATATACCCCCACACACACACACCCCTACACCCACACACACATCCACACACACCACAGCGGTGTTTATTTTTACATTCACAGCCAGAGGCATTTCAGTAGGTATTTCTCAGGAGGGAAAGTAACCCTTTTTCTCTAAGAAAAGGTCGTAGGTACTTACAATAGAATGTTGTAACCAGAATTTTTCAAATACTGTTGTTACCATATCACACTTTGCTTTGCTTCTTGTATTTGTAAGAAGCAGATTAAATGAGACTTTCACAAATGCCAATCTTTGCATTCCTGCCCCTTTGAGGATTATAATTAGAAAAGACATGAACTTAAAATTCCTCTCCTGTGAGAGACAAATAAACCAAGCCAGAAGCAATAGCAAATTACACCATTACTGATTAAAAAAAAGAATCTACTGTAATTGCAAAATTCTATATGGAATCTCCCTTAAGGAAAGAGTCTGCTTTTCGCATCTGATC |
| Pha76 | Osp108_B09 | GGGCGGACAACCCCCCCCACACACACACACACCGGCAACGGTACCAGAACTTCCAAAACCAGCTTTGAAAATGATTTCCAGAATAGGTTCTTTCTGGAAAAACTACGAATGTACTATACAAACGTTTTGAAACAAAACCTACTTCTTACACACACACAGATTTCGAAACATGCTGGAGCATAGTTCTACTTTGGGTTCGTATTTTTAAAAGTAGTACTTTATTTTTGTTTGAAATACACACGTCAAGTGCAGCAGAACGGTGCAAAAACCCTCACTTCCATAAGTGTAACAAACTCCAGAATCTAAATCTTTTTTAAAATAAGTCTCCAGTACTATGATTGCAGACACCCTTCATACACAAGTGCTGTGAAGAGACACAATAATAAAGTCTAGAGCCAAACAGTTCATCACTATTTTAACTGGCAGTTAAACCTGAAGATTGTTATGACAGTGAAATATGACCGACAACAAAACACGATC |
| Pha77 | Osp108_B12 | CTCCTGGTTGCCGAAACTGTGTCGTCAATTACTACAGACTGCTGTGTCACAATCACATTCATAAGCCTAATCAAGCTTCACCATATTCATATTTAGCTTCTCTTCCTCTTTGGTAACTTTGGAAAGCTACTCCACAATCTCAGTCCTGACCAAAAATTCCCAGCTTCCTGCCTCGTTTCCTGCAAAGCTACTTCTTATTTTCTTGTCCTCTGCCCAAATCAATGTTTCTGTTTATGAAAAGGCTATTTTCCTTCTCTCATAGATTTACAGAGGTCATTTATGTTCACAACTATCCTTTAAACAAAGGACTGATGCTCCTCCACACATACTTCCACAGTTCATTCTCTTGAAGATGAGGATTATTCCAGACAAGGTCTTACCAAAGCCTTGTCTGGCTGCTGCATTAATACTTTCATCTCTAAAAAGAGTTATACTGAAAAAATGCTTTTCACAGCTGCATTCACCAGTGGCTTATTGATATTACAGACCGAGACATTAAGAACTTCTCCTCCTTTGCTATTTTCACATGGTGACCTCATGGTTTGCAGCACACACACACACACACACACACACACACACACCCCCCCGAGTTTGACCTCAAACTTCCCCAAACTGCATCCCATCCCATCCCATCTCTGTCACTCTAGCTTCACCACCACTGGATC |
| Pha78 | Osp108_C01 | GATCAGTAAGTCTTAACGTGATGTAGTGAAAACAATTTTTCAGTCTGAGGAAGGTAATTCTTTCCATCCTATACAAGACTTCTCAAATTAATGTCTACCAGTGGACTTTCCTAACACTATGTTACTGTCTGTATTTTATTTATATATCGTGCATGTAAATATACATGTGCACACGCACACACACACACACTGTGTGTACTATATGTCAACAACTAATCAAAAATTGTTTGCCCACAAGCATATGTGAAAAATTCATCTAAATAATTGTACATTTAATTTTTAATGAAATCCCAGTAATCCACTCAATTTACCAAACTCCTACTTAATATGAAAGTGGCATGCACAGAGATC |
| Pha79 | Osp108_C04 | GATCGCTTCTGCTTCTCTGGCTTTCAGTTTCTCACCACTCCAGCGTAACGATTGCTTTTGTGCGTGTTTGCAGTTTACCAGCCCGGCAGCATTTGCCCTTCTTTCTCCGGCTCTCCCTAACGCGGGGGGGCACCTCCCGGAGCCTCTCCCCAGCAGGACGGCTCTCACGTAACACACACACACACACACACACACACACACACACCGGCCGAATGACACCCCCAAGCTTCCCGGGTACGC |
| Pha80 | Osp108_C06 | GATCCTGACCATCAGAGATGATAGGAAAAAGTGCAGCACCATTAGCTGACTGTGGGACTAGGAAGAGTGAATCTGAGTATCTCAGTATAGCTAAATATAGGATTGAAGCACTTATAGAAACAATCAGGTGCATTAGAAATGTTCAAATGTATATATGCATCAATCTGCCTTTAATGTCATTTCATCTGTTTATTTGTCAACCCCTTCCCTTATTTGTCATCCCAAGCTTGTGGTTCAGTGAGCTTCTGTAACCCTGAGCATCTACAACCTTGTAAAAGCTACCAATTAACTGAGGTCATGTCTAGAACCTGTTCAATAATATATGTATGAATTTCTCTCTCTCTTTCCTTCTTTTTGTCACTTTCTCTCCTTCATCTGTGCCTTAAATCAATGGAAGTTCCTAAAATTTTAAGAAGTCACATTCCCTACACCTCTGGAAACACCAATTATTTCTATGTGTTACTTTGTACACCTTGAGAAATCCAGAAACAAGAGCAATACATGCATGAAATGTGGAGTCCCAAAAGGCTTCTTTGATGTTCTTTTTTCCTGATC |
| Pha81 | Osp108_C07 | GATCATCTAGTCCAATCCCCTGCCAGGGACAGGGGCACCTTCCACTAGACCTGGTTGCTCTAAGCCCCATCCAGCCTGACCTTGAACACTTCCAGGGATGGGGCATCCACAACTTCTCTGGGAAACCTGTTCCAGTGTCTCACCACCCTCATCGTAAAAAATTCTTCCTTGTGTCCAGTCTAAACCTACCCTCCTTTGGTTTAAAACTGTTGCCTGTTTTCCTGTCACTACAGGCCCTGATAAAAAGTCTTACTCTCTCTCTTTCTTATAAGCCCCCTTTATATATTGAAAGCTGCAATAAAGTCTCCTCAGAGCCGTCTTTTCTCCAGGCTGAACAACCCCAACGCCCTCAGCCTTTCTTCACACGAGAGTTGTTGCAGCCCTCCGATC |
| Pha82 | Osp108_C08 | GATCTATGAAAGATGCTGCTGTTATTGTTGGGGTGTTAGTTGCGTTAAATCATGTTATTCATTCCTGTTCAATGGAGTGGCTGGATTTATTTTTAAAGATTTGTCGTTTACCTCAGGATGTCAATTAATTCAATTCCCAATTGCTTATTTGTGCTTCGTCCCTTTGGTTCATTTCGGTATGTGCAGGAACTTAAGAGGACGGAAAGGGGAAAAAAATGTATTGTTCCAGCTTGGTTTCCAATTTGTTATTTCAGTCTAGGTGAGAAGGCCAATCTGCAGTGATTCAAATACCTGCAAGGTGAGAGAGAGATGGGTGTTCTGGTGCCAAGAAAGCAAGCCCTTTTGCTGAGCAGGGTCTAGTTTGTCTTATTAACAGCTGCGTTTTCAGCATCCTTGAAACCTGAGGAAGCCATGTATGGGGATC |
| Pha83 | Osp108_C09 | GATCCCTTTCCAACTTCACCATAGCAACCCCCTTCAACAGAGAAATGGAGTCATTTCTGGGTGCGATGGCTGGCCTACTAAGCAGCATGAAAAAACAAGCGTGGGCTGATGCCTGCTTTTACAACACTTGGGTGGGTTCTTCATCCTCCTTCCAGTGCATGCACAACTTCCTTTTGCACAGGTCTACACACACACAGAGACTGAGTGCACTGTCAGGAGTCTAATTTACCTTAGAAAGCTCTACTGGGGTTAGATGTAAGGAATGGGATTTTCATTTTCACTTGTCTTCATTTCTCTGAAGGGCCCTTCATACCAATGTTTCCACAGCGCAAAGAGGCCGTCAGCACTGGCACTGCACCCACTCTGAGGTGCATTTATGCTTTCTGGATATTATAAAAGTGTCTTAGAGTAAATGAGAATTGGGGCTGTCTCTGCTTCTGACAACACTTAGACTCCCAAACTATGATC |
| Pha84 | Osp108_D01 | AGCTTGCTCTAAATGACTTGCATTACAGTGGAAGAACGCTTTAGCTGTGGCTCATCAACTCTTGAACTCTGCCCTTTCCTTGTTTTCCATTTGGGCCTTGATTTTTTTCTTTTTTTCTTTTTTTTTTTTTTTTTTTTTTTAGCTTTTAGCAAAACTTAAATTTTTTGAAAAAAAAAAAAAAGGGGGGGGAAATTAATTTTAAAAGGGGAAAGGAGGGAAGGGAGGGAAAAAAAGGAAAAAAAGGGAAGGATATAGAAAAAGTTGTTATAAAAAAAAATTTTTTTTTTAATTTTTTGGACGGTGTGCTTGAGCTGATTTACAAAGCCCAATTTATGGGATATTTTTTTCCTATGTATTTTTGTTTCCTCCCTGAAAAGAAATGAAAAGAAATGGGAATTTAAAAAAAAAAAAGTTGTCTGAACTTTTGATC |
| Pha85 | Osp108_D08 | GATCTGCACAGCCCCAGACCTGCTGACTCCATCAATGCTGTGCACACAGTATTGCCAGTGAGGGGACCCCTGTCCTGCCTTCTGTGCTTTCTCTTTCTCTCTTTTATTTTCTCTTTCTTTCTCTTTTTCTCTCTCTCTTTTACTTTTTCTCTTTCTCTACAGGGACAGAGACACCTGCAGTTTTCTCCTCACATCGAGGAAAGACTTGATC |
| Pha86 | Osp108_E02 | GGTACCCGGGAAGCTTGGACGAGCTTCCTGAAGACCTCTTCAGGCCTGAGGTCCTGAAGAGCTGAAGGTCTTGTCAAAATCGTGACGAGCCTTAACGATGCGCATACTGCAAAAGTTGAGGCTAGTTCTCCGCCACAGTCGTCCCAAGCTTTGTAAGTAACGCTTGTAAGTTGTTTGCCTGCTCTTCCTAAAGTCTGCACCGTCTTTTCGGCGTTATCCTCCCCCCGACGCCCCCCCCCACACACACACACACACAAACTTTGATC |
| Pha87 | Osp108_E03 | GATCTATAGCTCCTGGCTGGACAAAAGATTACCATTTAGCAGTGTAGCCAGGCTTGACTTAGAAGATACCACTAATCCCCTGTCTGCTTTGTCCAGTGGTTAATTACACACACTGACTAATTACATTGCATTTTTTTCCAGTGATAATTTCTGATGTCCTGTCATTGGATTCCTTTCAACACTGACTGCAGACTCTTTAGCTATATTAAAGCAATCTTACTCTTTAGGTTTTGGTAACTTCATTTTTCCTGCTTCTATATGATGTTTCTCTCTCTCTCTCTCTCAAAAAAAAAAAGATATTCAACAGCTACTTTCACAGCCTCTGTGAAGAGCTAACTAGAAGCTGAGTGCATATATGGTGATGCTTATTTCCAGTTCTCCATTTGGAAACTTAAACTACCATGTTAAGTAGCTTCACTTACATCTGCATCAGTCATGATC |
| Pha88 | Osp108_E04 | GATCCCACTACCTCGCTGCTGAAGTTAGTGACACAATGATGCGTCTGATAGCAGAGATTTGCATGTCTCAGGGTTGTTAGTGGTGATGGAGCAAGTGGCAGTGGTATTGCCAAGCAGAAGTGACAAGGTACCATGGCTTGGGTTTCTTTGGTCATTAAGAGGTAAAAATTTTTTTGAATGAACAGGAATAGAAGAGCCGTATAGACAGGCACACACACACACACATATGTAAATTAAGTTCTCATTATCCTCAGGATTCCTGTTGCACACTTAACACTGTTCTCAGTCAGTACAATTTCCAGTGTTTTAAAAAATTATATGAAGTTTGCTGGGATTATCTGGTTTATGATC |
| Pha89 | Osp108_E05 | GATCGCAGTCCTGAAGTCAGGAAGTGCATGTTAGATGCAGCTCTTTCAACGCTCAACACTCATGGCAAAGTAAGTCTGAATGCTTCTCTCTAGCAGTGGGAAAAAAGTGGGATGAAAATGTAGGTACTGTGTTGGAGCCCATAGATTTTTGTAATTTTTTTTTTTCTTAGAGGTTCCTGTTACTTGTAATAGTTAAAGAACTTTCACGTAGTAGGAATCTGTGTGTGTTTGGAGTTGTTCTGTTAGTGACTAATGTTGACCGAGTGTAGTTTTACCCTGTCATTGCAATAAAAAATAGCGCTGAATTAACTGGAGTTAAATGGTATAAAACTAGAGTAATAGAAGTCAAATCAGACCACAGGTCTTTAATTGTGGGAAGGTGAGTTTTCCCTTTCAGGTCTCATGCAAAGGAAAACATGAAAGCTTGCTGATC |
| Pha90 | Osp108_E09 | GATCAAAATATTTCCCTATTTAATCTCAACAAAATACACACAACTTAATTTTGTGTTTAAATATATGCATAGTTTAACAAACATTCTTAATTTATTCACTGCTTATCATATTTTAGATGATGTTTAAACAACACTACACACACACGTGCATGCAAGCATAGACAAGGCTGGTTTATTAGTCAAGAAGAATTCACCTGTACACAGAAATTATTTCTAATCAACCACTTATTTTGAGGAGTAAAGTGTCAGGTGCAATGCACTCCCACTTGAGTTTGTCAAACAGGTCCTTACTTAATTTTTTCAGTCCTTTATATGTTCCCCTTTAGCCAAAGCATTACAAGTTTCTGAGTAGATACAAATGAAGAACAAGTGTATCAAAATATACAGAGACAGTTCCCTGCTATTTAACGAGACAACAGTCAGTGATTGTTTAAATAGCACAATTTTAAGTTGCGTTAGAAAAAAACTATATCAAGTTATTATTTCAGGGAGGAAAAGAAGGAATGGTCTTTTCCTTTAAACAGGAAAATGGCTACCACAAACTTCGATGACTAGGAAGTGGTATCCCTTGCAAACATCAGGCCAGTCCTGAAGCTATTATTTATATGAGTACAGGTGAAGTCAAGTCTGACAAGTCCTTGGACCCCTTCCCACAAGCAAAGGTTTGCAGGATC |
| Pha91 | Osp108_E12 | GATCCCAGCCCCTTTATCAGGCACTGATTTTTCGCTTGTCTTCTTCAGGCATTTTCCCGTACATTTTGTAAAGGGCCACCTGAGCAGCTGACTCTTCTTCATTCCACTCTGGAGGCAAAGTTCTATTAAGTTCTGCTGAAGATGGCAAAACAGTAGAAAAATAAGTCCTTTATTGAAACTGTATGTTAAATTTCTTCTTTGCAGTCTTTTAATTTTTTTTTGTCATGTAAAACCATTGTTTTATTGGACTGTGAAGTGGCTTTATCTTACTCATTAGTGTAGCTGTGGTTTTGTGTGTGTGTATACCTGAATAAGATC |
| Pha92 | Osp108_F06 | ACACACACACACACACACACACACACACACACACACACAGTCTCTGCAGCTCTCCCAGCCCATTTCTTTCTGTTTTTATTTTTTCTGCACCCTGATTTATAAGGATGTGCCCTATCTTCATGTCTCAGGGAACACATAGGACTTTTAGTAAACATACACAATGCAGTTCTTTTTTTCCCAAGACTCAAAAGGAAAAGGCTCCTCCCCAAAGATAAACAACCCACCCACCCATTGCCTCAAGCAAATACATTGAGGAAGAGATGAACGGCTTTGCATTGTGCCCTGTAGGTCTTCAGGCAGCCGCCTTCTCCGGTCGAGAGCTCCATTGGTGAGGGCCATCTCTTGGGAGTGCCCTGCTGTGGGTCACCCCAGATGAGGTCTGGGACTCTCAGACCCAGCAGTACATGATGCCTGGCTTCTCCCTTACCCCCTTGCCAGAGAGACAGTCATTTTTCTACCAGGTGATAAGAATGCCCAAGGAGCCAGACGTTACAGGCTGCGCTCCATGGTCTGCAGGAGCTGATC |
| Pha93 | Osp108_F08 | GATCACAGTGACAGCTACAGTCGCATGAAACTTTCAATCAGAGCAGAAAACAACAGGGGCATAGCAGGAGGAAGGAGAAGAGAACAAAGCAGTGGCTCCTGCCATACTGACATCCCTTTGAAGGACTGTGTTGCTGCCAGGCAGCCCTCTGTGAGAGAGGAGATGTTGCTTGGAGGGGAGAGGGACACTACTTGTCTGCTTCAGGGAGGATGCTGTGAATCTCTGTGTAAGGCATGCAGTCCCTTGCTGGTTTCAAAGTACATTTTCTCTCTTTACAAGTGTGCACTCCTCCCCCATAAAACTGTTTATTTTTGTGAAAATTTGAAGTCTAGCTCAAATACAACCAAAGCTAGTTAAGATTTTTTTTTTCTTTTTCGCCTTAGGTCAATAGGGAGAGAAAAAGAGAGAGAGAGAGAAACATGTTGGGTAAGCTCCTTCTTTATCTGCAGCAATAAAAAATTTAAAGCAAAGCTTGGCTGAACCTCCAGTCCAAATGTCACCTTGGCCCTAATATTTCACTTCTTGTTATGAGTCTAATTTGTATTTATTGGTGCTGCACAGTTCTACATTTACACAGATGCTCTGGCTTCTTATGTGTAGGATGATC |
| Pha94 | Osp108_F10 | GATCAAGGTTGGAAATACTTTTCTGCAGCTATCTTCAGTAATGTATATACACATGTATATATATACTTCCAGGACAGCTATATTTCAACTTGTTGTAGACAGCAGTTTGTGAAACTAGAAGAGAATCATCTGTCTTTTAGCAATTCAGTAATTCAGTGCCAGATATATTTTTTGGCTCTCACGGAAGAACGAAATGCAGCATACAGACACACACAGAAATTACACACACACACACGTGTGCACACACACACACATATGTTGACTCTAGAAGGGAAATATGAAAGATGTGAGCAATGGAACAGATGGGCCAGTTTTAATAATATAGGCATTTTCAGCAAAGCAGTCTGCCTCCGTACTTATACCACTGAAGCTTTATTAATGTTGTAGATTATATCATTCAGATC |
| Pha95 | Osp108_F11 | GATCTTGTCTTGGAGCTGGGGAAAAGGCTGAATGGCGTTCTCAAGTCCATTCCAGCCCTGAGAGGCCATAAGTATATGCATGTATATATGTGTGTGTGTGTGTGTGTAGGTATATATATGTGTGTGTGTGTATACATGTATGTATTTCCACACTTCCGGTGACAAGCTTTTGGCCTCTGTGGTATTTTGTGGCGGTGAATCTTGTAGGCTAATTACACATAGGTTTGCTGTCTCTCGGTTTCATTAAATTTGAGTTTCAGCTCCGTTTAATTCAATGTCCTTTGTGCCTCGAGTGGATC |
| Pha96 | Osp108_G02 | GATCAGTATAAAACTATTTTTCAGCTAGCCATTTTGAAGTAGCCATGGGACCTGCTTTCAGTTGTACTTGACTACAGTTCACAGTTCTCCCCACACACACACCTTCAGCTGTTAGAATTGGACCTTTATACATGGTTGCACCTGAAATAATTTTACCAATGCTACAGCAGGACAAAAGGCTTAGTTAAATCACAGTAGCTGCTGGGTGTTTCCTTACATTACAGCTATTAACAGGGGTATTTAATAACAGTATTGGGGTGTATCTGTGTGTCTTGTAGTAAAAGACAATAGACTCCAGCTACCAAAATATGTCAAGTGCTAATGACACTTTCTATTTGCATAATACAGATAATATATAATATAAAGCACAAGGAGCACAGTGTATCTCTCCTGTAATGAAAGGCTGAGAGATC |
| Pha97 | Osp108_G06 | GATCAATTTGGCTTTGTCAGCAGTATTGTATGTCATAGCCTGGAGAATGATGAATTGGTTAACATGTTTGATGTCTGTATGCAATATGCTTGATGCCCCATCCAATGTGGATATCCCCAAATCAATTGATGTTCTGAAACGTCATTAATTCTTCTTTTATATAATCATTGTAGTTGCCTTTCATCCTCTCTCCTTCTCTCTCTCTCTCTTTTCTTTTCCTTTTCCTCTTCCTCTTCTTTATCATGCTACCACCAAGTGGTCCTGACGACCATGTTGAGCCATGCGGTTGTGTAAGAGTCGGTCAGAAAAGCAGCATTGTCTCACCAACGTTATCAGGACGGAATGTGCAGTACAAGGCTCCTGGATGATACCTATCTAGAGCCGTTAGAGATAACTGCATAGTTTCTGTCAAAAAGGATGGATTGCAACTGTTGCCATAACATTGATGAAGAAATCGTGAACTTCAGGCAAACTTTGCTTCATTATAATACCAAAACACACCTCCTTGTCGAAGGCTACCCACCTCCAAGGCTTGCCACGCCTCGGACACTGACCCTGCGCCTGCGTGATAGCCTCGCTCGAGAAGGGCGGAGATATGCTAAGAAGGGCAGAGACTCCTGAGGCAGAGGTGGAGCAAGGTGTGTTAAAAGATGACTTCTGAACGACGGAAATTTGGAACTTCCCCCATCAAGGACCACAACGATC |
| Pha98 | Osp108_G08 | GATCCAAAGTGGAACTCCAGCAATTAAATAGTTAATCACTCTGTGTGTGTGTGTGTGTGTGTGTGTGTGTGTGTGTGTAAAATTCATGTGTATCTGAAACCACCTCTCTTATCTGTGTAATGGTTTGGTTTGATGTAAATATTTTTAAATGTGCTTTTACATTAGGCTGGTTTTTATACAGTGTGTGAGTGGAATTGGTTTGTGGTTAACTGGGCCTGGACTTTATATATTTGCTCCATTCACCTCAACGTTTCTGAACTGATACGGAAAATTTCTGATC |
| Pha99 | Osp108_G09 | GATCCAAGGTGAGACCTTTTGAGCTCTCTGGGACCGCAGCGGGCTGTGTGACCCAGCTGGGACGCCTCTCAGGGTAGGTTTCACAAGGATTGAAAGGCAGTCTGTCAACGATTTCACGAGGACTTAAAGGCCTGGTTTCGTGAGGTTTACTGAATGTCCGTTGATGAATGCTGGCACATAAGCGTAATTCATGAAACTCACGAAAGGGAAATTTTAATCATAGGTTAAGCAGTGTGTGTGTGTGTGTGTGTGGGTAAAATTAAAATTTTGTTCAGAACTGTTGTATCTGTGCACGTGCATTTGCAGTTTGGTTCAGAACTATTTGCCTGTCCCTGTGTGATTTGGAACCGTTTTCTCTGTCTGCATGTGTGTGATTCGACTTAACCTCCGAGTGCAACCGTGCTGTAAGTGTCAGGTGATC |
| Pha100 | Osp108_G10 | GATCTGAGTACAGCAAAAGCCCTGGTTTGAATAGTGCTACATAGGCAGCATCTAGATGTTAGGGCTCCTTGAGCTCCCTGAGTAATTCACACACACACACACACACACCAGCCATATTAAATTAATTTTGGAAGACGTTTGCAGGAACATACCAACTTTTAAATTTGCACCAATCGTGTGCTATTTCTACTCCTGCCACAGCGGACCTGCCGAGTTAAGTTTTGGCCATTTAATTTTTCTAAAGAGCAGCAAATGGCAGAGCGAAGCCAAGGCAAGGTGCAGAGCTCCGTGTGAAGAGACAGTGTTATGGGAGCACAGCAAGAAGCAGAAACGAAGCTCTGTGTAAATTGATC |
| Pha101 | Osp108_G12 | GATCTTGAAGAAACTCAAGTTATTTTAGTTGGGGCTAATCTATACCAGCTAAGTATTAAGAAATTATCCGGTATTCACTGTGTCACAGAGAAAACAGATACATTATTCTACCTGTTAGGGTGATTATATTTCTCTCTCTCTGTGTGTGTGTGTGTGTGTGTGTGTGTTCTAGGATGCTTTGGATGCTATATTAATATTTATACTATACCAAACAGATAATGGATTAGTGTCATGTTTCATGATATAGTTAATAATTCTCTTAGTGGGTGAAGAGGAAGTAACTGATC |
| Pha102 | Osp108_H03 | GATCTTCAAATGTACCTTGGAGCCTAACTTCTTGTTTTTCTAAGTGAGATGTTTCCTAAGGATGTTGGAGAGACCGAGACACTGGTTCTTACTTGCCTCCAACACAACAGCGGCACTCTAGTTTTTCATTTTGCACTTTCCTTTTGTGTGTGTGCGTGCGTGTGTGTGTGTGTGTGAGAGAGAGAGAGAAAAGGAGGTTTGGTCATATTTTCCATTCTTTCTAATGCATTTTGAGAGCCTCAGGGAAAAGTGCTAGGCAGGAGCAGTGCCATTACAGACAAGCTTCCACATTATACACAGCTAGAGATGACAAAAATAGCACACAACTGGGACCACAGCAAGGACTGGGGTAAGGAATGGTCAGGGAAAGATAGGGAAGTCCCTGGGGTTTAATGGCTGAGTAGGAAGAGATGTTGAGAGCACTGATC |
| Pha103 | Osp108_H04 | GATCTATATGGGACTGCGTTCTCTAGGTTTATTAGTACAGGAGCCACAAACGTAAGAAGGGGATGTTTGAAAACTTGCACCCATGGGCATGGATGGATGGATGGATGATAAGGTTTAACTTTCACAATACAGTGCTAACTGATGACAACTGATTAGGAGGACAGCCTCTTTGTAAGAGGTGAATTTTTATGTGAAGTCAAGTCCTAGTACCACCCTTTGCAGGGACTTTTATTCTACTTTAGGTTCTTTGTGTGTTTGCATGTGTGCATGCGCGTGTGTGTGTGTGTGTGTATGTGCATGTCTGTGGGAGCTGGCTGATGTTGCTGGGACTGAGTTCTCCTGCAAGCAAATAATTCAGCAAATGTGCCTGTTGTCTTAAATCAATACACAAATGTTAAAAATCCCTATTTGATGGATAATTTAGTACTATTCTCACAGCTCTGGAAATCTACCCCAAAAAGCAGAGTTTATTCCTTGCCTTAATTTGTTCATTTTTATTAAATAAATTTTTAAAAAAGGAGATATAATGATGTCATTTAACTCCAAATGCATTAAGAAGAAAAAGAGGAGAAATAAAAACAACCACAGTGTTTTCCTTAATTTTTCATCTTGCTCTGTCAAAACAGTCCCCAGCTTTCATGATC |
| Pha104 | Osp108_H05 | GATCAGGGTTATCAGCTTAACCCAGGGAACTCCTTTGGGTGATTAATCTTTCAAAAATGAATAGAGATAATCAGTACAGAATATTACTTTTCATATAGTTCCATTTTCTGAAGACTTTTTGTTGTGTGCCCACTCCTATCTGTTCTACTTTGTTCTTCACTTTCCTCCTATCGATGCTCTTAATACCACGCAACAGACTTCTAGGCTGCCGCTGTTCTTTTAATCCAAACTCCAGCTACAAGCAGCTGTTCTGTAGGCAGACTTTTTTTTATTAACTTCGTTAGGAACTATGCCGTATCATATTACCATCTTCTGTTCCCCGCCCGTGCAGCTGCAGTCATTGCTTTCCACAGAAGTCTTTACAGAAAGCCGTTAACTTCTCTCTTTTCTCAGTACATTTTTGTGTGTGTGTCCTTTGCTGCCCTTAAGTTATCTTTGCATTGTGAATAAGATTTTTTTATACTGAGTATTTCTGTTGGTTTAAACCAGTTCCACTGGCCCAGTTCAACTTGATC |
| Pha105 | Osp108_H06 | GATCCTAAAGTCTATAAACAAGTACTATTATTTAAATATTATTTCCTCTAACTGGGAGATTAGTCTTCCATAGTTACAGAAGCCCTGGATAAAATTAAACCTAATACTAAATATTAGTTTGGAAATGAATGTGCATTTTAAAATTGAATTGGGGTTCTAAAGCAGGCTGTAGCAATATCCAATCTCAGTATTTTTCCCCAGTGACTTGGACCCAAACAGCTTCAGTCTTTGACCTTTTTAAGCTGACATTTTCCTATCCCCTTTACCTGTTTGCTCTTCTGTGAATACCCAGTGTTTCAGTCATGATTGCTATTTTTGTGCGTGTGTGTGTGTGTGTTTCAATTCTTCATAGCAAGTTCCAGATC |
| Pha106 | Osp108_H07 | CTGACATTTTCCTCCATTCTTTTTTTCCTTGCAGGCCATTGCTGACAAAAAAATATTTCTGTTAGGTGTAGGTGTAAATTTCCATCGTGGTTAATATCTCCAGTCCTTCAGCAACAAGTAAACATGTAATCCCACTCTCTGTTCTCATATCAGGCCAAACATTCCCGTTTATTGAACCTTCCACAAAAATATCTCAGGCCGCTCTTTCCTGATTAAGTGATGTTTTACTCTTTCTGTATGTGCGCATACACACACACACGCACACACACACACTCACTAGGTGGTTATTTTGGACCCATGTTTGGATAAGGACCAGCTCAGTTCAACTGAACGTCTTCCTCAATATTCTGTTCTGTTTGCCTTAATAAATTGGGGGTAGGATGGCAGGGGGAAATTCCTCAGGATTAAGTACTGGCCTCTCCTTGAAAAATGACTGAGTTTCTCCTTTAAAAGCTCAATAAATTTCTCCAGTAGCTCTAATCATGCTTGCACAAATTTTGAAACCAGATTTTTACAAATTGTGTCAAACTTTATAGTGTTAAATCCATATTGCATACATTTCTACCAATTCTATACATAGCTTATGCCTATAGGATC |
| Pha107 | Osp108_H12 | GATCTGGCTGATGTAGAGCTTTAAACAGCTTAAAAAGAACAACACCCCCCCACACACACACTTATTTATAGATGTATCTAAAGTGAACACTATGTTTCTTAATTCTTCCAAAACAATGAAATACTAACAAAAAACCTAAACATTCTTTGCACCTCAAAGAGCTCTCCTGGGGCATGTTTTCTAGAAAATGAAGCGGCCATCTACTCTGTAAGTATTGCAAGTGCTCTGTTCAATTGCGGCCCCATCGATC |

* Locus Pha01–Pha37 were submitted to the ENA, European Nucleotide Archive: see <http://www.ebi.ac.uk/ena/data/view/LN829364-LN829400>. Loci Pha01–Pha26 and Pha38–Pha107 were Sanger sequenced (bidirectionally) whereas the sequences of Pha27–Pha37 were obtained using a MiSeq.
