# Supplementary material for: A multiplex microsatellite set for non-invasive genotyping and sexing of the osprey (Pandion haliaetus)
Source: Conserv Genet Resour. 2015 Oct 29;7(4):887–94. doi: 10.1007/s12686-015-0497-4 (PMC4657739; doi:10.1007/s12686-015-0497-4)
Supplement: Supplementary file 2 — Supplementary material 2 (DOC 57 kb) [file 12686_2015_497_MOESM2_ESM.doc]

**Dawson DA**, Kleven O, dos Remedios N, Horsburgh GJ, Kroglund RT, Santos T, Hewitt CRA (2015) A multiplex microsatellite set for non-invasive genotyping and sexing of the osprey (*Pandion haliaetus*). *Conservation Genetics Resources*.

**Supplementary Figure** Chromosomal locations of osprey (*Pandion haliaetus*) microsatellite sequences in the zebra finch (*Taeniopygia guttata*) and chicken (*Gallus gallus*) genomes

**Tgu6**

**Footnotes**

Tgu, zebra finch chromosome name.

Gga, chicken chromosome name.

A location could not be assigned for seven loci: *Pha01*, ***Pha28***, *Pha32*, *Pha34*, ***Pha35***, ***Pha36*** and ***Pha37***.

Loci that are bold and underlined in the Figure and Footnotes were included in the multiplex sets (see Table 2).

*Pha01* displayed homology to chicken W and Z chromosomes in the NCBI sequence database but also to multiple other chromosomes in the ENSEMBL databases so we cannot be confident of its genome location.

*Pha12* was assigned to the TguZ chromosome. The genotyping of this locus also suggested it was Z-linked. When genotyped in 21 female and 27 male ospreys, all females were homozygous yet a proportion of the males were heterozygous.

**Pha29* (chromosome 6) also displayed an additional hit to the zebra finch Unknown chromosome (this is regarded to be an artefact of the assembly process).

Six loci in italics failed to amplify reliably with the primer sets tested (new alternative primer sets could be designed and tested if required): *Pha01* (ZW/multiple chrs), *Pha08* (chr. 7), *Pha22* (chr. 8), *Pha24* (chr. 1), *Pha26* (Z chr.) and *Pha32* (no hit).
